# Thymic stromal lymphopoietin modulates T cell response and improves cardiac repair post-myocardial infarction

**DOI:** 10.3389/fimmu.2024.1467095

**Published:** 2024-12-05

**Authors:** Xuhong Wang, Qi Zheng, Lingfeng Zha, Lingxue Zhang, Mingkai Huang, Si Zhang, Xuzhe Zhang, Qinlin Li, Xinglin Chen, Ni Xia, Min Zhang, Bingjie Lv, Jiao Jiao, Yuzhi Lu, Muyang Gu, Fen Yang, Jingyong Li, Nana Li, Xiang Cheng, Zihua Zhou, Tingting Tang

**Affiliations:** ^1^ Department of Cardiology, Union Hospital, Tongji Medical College, Huazhong University of Science and Technology, Wuhan, China; ^2^ Hubei Key Laboratory of Biological Targeted Therapy, Union Hospital, Tongji Medical College, Huazhong University of Science and Technology, Wuhan, China; ^3^ Hubei Provincial Engineering Research Center of Immunological Diagnosis and Therapy for Cardiovascular Diseases, Union Hospital, Tongji Medical College, Huazhong University of Science and Technology, Wuhan, China

**Keywords:** thymic stromal lymphopoietin, myocardial infraction, heart failure, inflammation, CD4^+^ T-lymphocytes

## Abstract

**Background:**

The inflammatory response is associated with cardiac repair and ventricular remodeling after myocardial infarction (MI). The key inflammation regulatory factor thymic stromal lymphopoietin (TSLP) plays a critical role in various diseases. However, its role in cardiac repair after MI remains uncertain. In this study, we elucidated the biological function and mechanism of action of TSLP in cardiac repair and ventricular remodeling following MI.

**Method and Result:**

Wild-type and TSLP receptor (TSLPR)-knockout (*Crlf2^-/-^)* mice underwent MI induction via ligation of the left anterior descending artery. TSLP expression was upregulated in the infarcted heart, with a peak observed on day 7 post-MI. TSLP expression was enriched in the cardiomyocytes of infarcted hearts and the highest expression of TSLPR was observed in dendritic cells. *Crlf2^-/-^* mice exhibited reduced survival and worsened cardiac function, increased interstitial fibrosis and cardiomyocyte cross-sectional area, and reduced CD31^+^ staining, with no change in the proportion of apoptotic cardiomyocytes within the border zone. Mechanistically, reduced Treg cell counts but increased myeloid cell infiltration and an increased ratio of Ly6C^high^/Ly6C^low^ monocyte were observed in the ani hearts of *Crlf2^-/-^* mice. Further, TSLP regulated CD4^+^ T cell activation and proliferation at baseline and after MI, with a greater impact on Treg cells than on conventional T cells. RNA-seq analysis revealed significant downregulation of genes involved in T cell activation and TCR signaling in the infarcted heart of *Crlf2^-/-^* mice compared with their WT counterparts.

**Conclusion:**

Collectively, our data indicate a critical role for TSLP in facilitating cardiac repair and conferring protection against MI, primarily through regulating CD4^+^ T cell responses, which may provide a potential novel therapeutic approach for managing heart failure after MI.

## 1 Introduction

Myocardial infarction (MI), primarily caused by thrombotic occlusion of the coronary artery, is the primary cause of high morbidity and mortality rates among all cardiovascular diseases (1–3). MI typically has catastrophic consequences and requires immediate emergency intervention (4, 5). In recent years, significant advancements have been made in managing risk factors and implementing primary reperfusion treatment for MI (2, 6–8). Despite this, heart failure after MI presents a considerable clinical challenge for individuals who survive the initial event (9, 10). Therefore, identifying new treatments and strategies for post-MI heart failure is imperative.

Thymic stromal lymphopoietin (TSLP) is a component of (IL)-2 cytokine family (11) and an IL-7 paralog (12). The functional receptor of TSLP is a heterodimer consisting of two subunits: the TSLP receptor (TSLPR, a common γ-like receptor chain) and the IL-7 receptor α chain (IL-7Rα) (13). The initial discovery of TSLP occurred in the culture medium of thymic stromal cell lines, providing support for the proliferation and maturation of B cells (14). Currently, TSLP plays a pivotal role in the pathogenesis and development of diverse diseases as an “alarmin” in response to endogenous and exogenous stimuli, including allergic disorders, host defense against infections, malignancies, metabolic disorders, and chronic inflammatory conditions by regulating inflammatory responses (15–19). TSLP also enhances type 2 inflammation, thereby promoting allergic responses (20, 21). The monoclonal antibody-tezepelumab, specifically designed to target TSLP, received approval for the management of severe asthma in 2021 (22). In thymic tissues, human TSLP is involved in the differentiation and homeostasis of Tregs via CD11c^+^ dendritic cells (DCs) (23). During MC903-induced skin inflammation, the TSLP/TSLPR pathway in DCs contributes to the expansion and proliferation of local Tregs (24). Furthermore, in atopic dermatitis caused by Mi-2β deficiency, TSLP can directly act on local Tregs in the skin, promoting their proliferation and differentiation into effector Treg phenotypes (25).

MI triggers a robust inflammatory response, and the role of inflammation in heart repair and ventricular remodeling following MI has attracted significant attention (26, 27). The release of danger signals from necrotic cells activates immune pathways, triggering a strong inflammatory response that aids in the removal of necrotic cell debris and scar formation. Both excessive and insufficient inflammatory responses lead to adverse ventricular remodeling and poor cardiac repair (28).

Recently, a clinical study revealed that patients with acute MI have higher plasma TSLP concentrations than those with unstable angina (UA) (29). However, the precise role and fundamental mechanism of TSLP/TSLPR in cardiac repair and ventricular remodeling following MI remain unclear. In this study, we investigated the function of TSLP/TSLPR signaling after MI.

## 2 Materials and methods

### 2.1 Mice

Male C57BL/6J mice aged 8–12 weeks were acquired from Beijing Vital River Laboratory Animal Technology (Beijing, China). Cyagen Bioscience INC (Nanjing, China) generated TSLPR knockout (cytokine receptor life factor 2 [*Crlf2*]*^-/-^*) mice with a C57BL/6J genetic background. All animals were accommodated at the Animal Care Facility of Tongji Medical College, where they were provided with a standard diet and maintained at 25°C. Furthermore, they were housed in a room with a strictly controlled light-dark cycle of 12:12 h. The studies involving animals were conducted in strict compliance with the guidelines issued by the National Institutes of Health and were duly approved by the Animal Care and Utilization Committee of Huazhong University of Science and Technology in China (Institutional Animal Care and Use Committee Number: 3775).

### 2.2 MI model establishment

The MI model was established by permanent ligation of the left anterior descending artery (LAD), following a previously established protocol (30). Briefly, the mice were anesthetized by an intraperitoneal injection of 1% pentobarbital sodium (60 mg/kg), followed by oral intubation and subsequent connection to a ventilator designed for small animals. After removing the hair from the surgical site, the surgeon proceeded to open the chest cavity and expose the heart. Subsequently, the LAD artery was ligated using a 6-0 suture. In the sham group, mice underwent the same surgical procedures as those in the experimental group, with the exception of LAD artery ligation. Finally, the chest cavity was closed and postoperative monitoring was performed.

### 2.3 Western blotting

Whole hearts were harvested from the mice, and the atria were removed as previously described (31). The ventricles were washed, dried, weighed, subjected to protein lysis (Servicebio, Wuhan, China), and centrifuged (12,000 rpm for 10 min). After centrifugation, the supernatant was collected. Protein levels were measured using a bicinchoninic acid protein assay kit (Thermo Fisher Scientific, Waltham, MA, USA). The samples were supplemented with loading buffer (Epizyme, Shanghai, China) and then boiled at 99 °C for 10 min. Using 12.5% sodium dodecyl sulfate–polyacrylamide gel electrophoresis, the proteins were separated (120 V for 1.2 h) and then transferred to a polyvinylidene difluoride membrane. The membrane was blocked using a 5% milk solution for 2 h, followed by overnight incubation at 4°C with either the anti-TSLP antibody (Abcam, Cambridge, MA, USA) at a concentration of 1 µg/mL or the anti-β-actin antibody (Proteintech, Wuhan, China) at a concentration of 0.2 µg/mL. The membranes were subsequently washed in Tris-buffered saline containing Tween 20 (TBS-T) for three cycles of 10 min each. Following this, they were incubated with horseradish peroxidase–conjugated (HRP-conjugated) secondary antibody (Proteintech, Wuhan, China) at a concentration of 0.025 µg/mL for 2 h, maintained at room temperature. They were subjected to three 10-min washes in TBS-T and then developed using an Omni-ECL™ Enhanced Pico Light Chemiluminescence Kit (Epizyme, Shanghai, China). The ChemiDoc MP Imaging System (Bio-Rad, Hercules, CA, USA) was used to detect and quantify the chemiluminescent signals.

### 2.4 Isolation of adult mouse cardiomyocytes

Cardiomyocytes were isolated from WT mice or the sham group on day 7 after MI using the Langendorff method, as previously described (32). Buffers were prepared as follows: liquid A consisted of modified Tyrode’s solution (Solarbio, Beijing, China) and EGTA tetrasodium salt (HUSHI, China); liquid B contained Tyrode’s solution (Solarbio, Beijing, China), CaCl_2_ (HUSHI, Shanghai, China), and glucose (HUSHI, Shanghai, China); liquid C consisted of modified Tyrode’s solution (Solarbio, Beijing, China), CaCl_2_ (HUSHI, Shanghai, China), collagenase, and protease (both from Worthington Biochemical Corporation, Lakewood, NJ, USA); liquid D contained modified Tyrode’s solution (Solarbio, Beijing, China) and CaCl2 (HUSHI, Shanghai, China). The perfusion system was rinsed with Tyrode’s solution and filled with liquid A after drainage. A 20-g blunt needle and 1-ml syringe (Jinta, Shanghai, China) were filled with liquid B, connected to the microscope arm, and adjusted to the best position. Mouse hearts were harvested, and the arteries and veins were quickly removed. The needle was inserted into the aorta and secured using 6-0 sutures for perfusion of the heart with liquid A. Next, the heart was fixed in a perfusion device filled with liquid C and subjected to perfusion circulation for approximately 7–10 min. The hearts were excised and digested in liquid C. The resulting cell suspensions were subsequently sieved through a 100-μm cell strainer (Biosharp, Hefei, China) and managed with liquid D to stop the digestion process. The centrifugation process was applied to the suspension at 20 x g for 2 min, subsequently discarding the supernatant. Pre-warmed cardiomyocyte medium (ScineCell, Carlsbad, CA, USA) containing CaCl_2_ was added to the supernatant. Finally, the cardiomyocytes obtained were extracted for further experiments.

### 2.5 PCR

#### 2.5.1 Real-time quantitative PCR

Cardiomyocytes and cardiac tissues were rapidly cryopreserved in liquid nitrogen and then stored at –80°C. The TRIzol reagent (Vazyme, Nanjing, China) was used to extract total RNA. The concentration of the extracted RNA was measured using a NanoDrop spectrophotometer (Thermo Fisher Scientific, Waltham, MA, USA). The complementary DNA (cDNA) was generated using a HiScript III RT SuperMix for q (+gDNA wiper) kit (Vazyme, Nanjing, China). The gene amplification process was facilitated using a custom-designed 96-well plate, and the PCR mixture was meticulously prepared using the ChamQ SYBR Green PCR Master Mix (Vazyme, Nanjing, China). The plate was centrifuged for 1 min at 500 x g. The plate was subsequently sealed and subjected to analysis using the CFX Connect Real-Time PCR Detection System (Bio-Rad, Hercules, CA, USA). Samples were examined in duplicate, and the relative expression of target genes was standardized to glyceraldehyde-3-phosphate dehydrogenase (*Gapdh*) using the 2^-ΔΔCT^ approach. The primer sequences for *Tslp* were as follows: TSLP-F, 5′-GGTTCTTCTCAGGAGCCTCTTCATC-3′; TSLP-R, 5′-AGGGCAGCCAGGGATAGGATTG-3′. For *Gapdh*, the primers employed were as follows: GAPDH-F, 5’-AGAAGGTGGTGAAGCAGGCATC-3’; GAPDH-R, 5’-CGAAGGTGGAAGAGTGGGAGTTG-3’.

#### 2.5.2 Qualitative PCR for genotypic identification

Mouse tails were collected and assigned unique numbers, followed by DNA extraction using the TIANamp Genomic DNA kit (TIANGEN, Beijing, China). Target sequences were amplified using the PCR system, and subsequent agarose gel electrophoresis was conducted to detect DNA fragments for accurate genotype determination. The following PCR primers were used for the identification of WT and positive heterozygous mice: Crlf2-F, 5’-GCTACAGTTCTTGGGCCTCTCGTT-3’; Crlf2-R, 5’-TCACCTGTGAGTCGAGTGGCGT-3’. The PCR primers for identifying positive homozygous or heterozygous mice were as follows: Crlf2-F, 5’-CTAGCGAGTGGACAGCGGTGAC-3’; Crlf2-R, 5’-TCACCTGTGAGTCGAGTGGCGT-3.’

### 2.6 Histological analysis

Mouse hearts were harvested and stored overnight in 4% paraformaldehyde at 4 °C. Subsequently, the hearts were paraffin-embedded and sliced into 3-μm thick sections for further analysis. Five to six sections from the apex to the base at equal distances were obtained. On the 28^th^ day after MI, the scar size and interstitial fibrosis were quantified using Masson’s trichrome staining (Biossci Biotechnology, Wuhan, China). Scar size was determined using the equation “(epicardial infarct ratio + endocardial infarct ratio)/2 × 100%” for each section and averaged from 5–6 sections, as previously described (33). Infarct thickness was measured at five equidistant points along the infarct scar in the papillary muscle section and then averaged. Interstitial fibrosis was measured at the papillary muscle level. To evaluate fibrosis, five random fields were selected within the border zone at 200× magnification. Using ImageJ software (National Institutes of Health, Bethesda, MD, USA), we calculated the collagen volume fraction by measuring the ratio of the blue-stained collagen area to the total tissue area (34).

For immunofluorescence staining, sections were processed according to a previously described protocol. To assess angiogenesis, one section per heart was selected and stained with anti-CD31 (eBioscience, San Diego, CA, USA). The area of CD31-positive staining was quantitated in five randomly chosen high-power fields located within the border area (35). The cross-sectional area of cardiomyocytes was measured using staining with wheat germ arginine conjugated with fluorescence (WGA-AF488) (Sigma Aldrich, St. Louis, MO, USA) (36). Terminal deoxynucleotidyl transferase dUTP Nick End Labeling (TUNEL) staining was conducted using the *In Situ* Apoptosis Detection kit (Servicebio, Wuhan, China). Slices were stained with rabbit anti-mouse TSLP, rabbit anti-mouse CD31, rabbit anti-mouse alpha smooth muscle actin (α-SMA), rabbit anti-mouse vimentin, rabbit anti-mouse actinin (all above antibodies from Servicebio, Wuhan, China), and goat anti-rabbit secondary antibodies (Servicebio, Wuhan, China). Prior to scanning and capturing representative images with a Nikon A1Si microscope (Nikon, Tokyo, Japan), the nuclei were stained with 4′,6-diamidino-2-phenylindole (DAPI) (Servicebio, Wuhan, China). Microscopic images were analyzed using ImageJ software (National Institutes of Health, Bethesda, MD, USA).

For 2,3,5-triphenyl tetrazolium chloride (TTC) staining, hearts were collected on day 1 after MI and cut into 4–6 pieces. All pieces were stained with TTC solution (Servicebio, Wuhan, China) and incubated in the dark at 37°C for 30 min, followed by capture of images (Nikon, Tokyo, Japan). ImageJ software (National Institutes of Health, Bethesda, MD, USA) was used for quantification.

### 2.7 Echocardiography

On day 28 post-MI, a Vivo 3100 high-resolution microimaging system (Fujifilm VisualSonics, Tokyo, Japan) was used for echocardiography. The procedure was performed by a skilled technician that was unaware of the treatment groups, ensuring unbiased and accurate assessments. Mice were anesthetized with 1.5% isoflurane, and long-axis images were acquired using the parasternal long axis (PLAX) view. In addition, three distinct levels were imaged through the parasternal short axis (PSAX) view in B-mode for comprehensive echocardiographic analysis: one near the apex of the heart (SimpAreaDist), one at the midsection near the papillary muscle level (SimpAreaMid), and one just under the base of the aorta (SimpAreaProx). At each level, the endocardial border was traced during diastole and systole. The left ventricular ejection fraction (LVEF), left ventricular end-diastolic volume (LVEDV), and left ventricular end-systolic volume (LVESV) were calculated from the digital images using a previously described standardized formula (37). The heart rate during echocardiography was maintained between 400 and 600 bp/min.

### 2.8 Flow cytometry

The spleen, peripheral blood, mediastinal lymph nodes (MLNs), and heart were collected from mice under both healthy and post-MI conditions. The spleens and MLNs were surgically removed, homogenized, and subsequently passed a 70-μm cell strainer (Biosharp, Hefei, China). Using Red Blood Cell Lysis Buffer (Solarbio, Beijing, China), we processed peripheral blood samples to isolate purified leukocytes for subsequent analysis. The hearts were minced and digested in a 0.1% solution of collagenase B (Roche, Basel, Switzerland), as previously described (34). The single cell suspensions were gathered and prepared through the use of a 40-μm cell strainer for filtering (Biosharp, Hefei, China), followed by purification of the leukocyte-enriched fractions using a 37/70% Percoll gradient (Sigma-Aldrich, St. Louis, MO, USA) (31).

TSLPR expression was determined on immune cells in the heart and spleen on day 7 after MI. DCs, neutrophils, and monocytes/macrophages were stained with BV510 FVD (BD Biosciences, San Jose, CA, USA) and FITC anti-CD45, PE/Cy7 anti-CD11b, APC-Cy7 anti-Ly6G, PE-Cy5.5 anti-CD11c, PE anti-major histocompatibility complex (MHC)-II, and APC anti-TSLPR (all from BioLegend, San Diego, CA, USA) at 4°C in the dark. CD4^+^T cells and Treg cells were stained with BV510 FVD, FITC anti-CD45, PE/Cy7 anti-CD4, and APC anti-TSLPR (all from BioLegend, San Diego, CA, USA) at 4°C in the dark. After washing, the cells were fixed and permeabilized using Foxp3/Transcription Factor Fixation/Permeabilization Concentrate and Diluent solution, along with Permeabilization buffer (10X) (both from Thermo Fisher Scientific, Waltham, MA, USA), followed by staining with PE anti-Foxp3 antibodies (eBioscience, San Diego, CA, USA).

To quantify the cardiac-infiltrating lymphocytes on day 3 and day 7 after MI, the collected cells were stained with BV510 FVD (BD Biosciences, San Jose, CA, USA), FITC anti-CD45, PE/Cy7 anti-CD4, PE anti-CD19, and Percp-Cy5.5 NK1.1 (all from BioLegend, San Diego, CA, USA) for 30 min in the dark at 4 °C. Next, the cells were washed thoroughly and stained with PE anti-forkhead box P3 (Foxp3) (eBioscience, San Diego, CA, USA) before being fixed and permeabilized using Foxp3/Transcription Factor Fixation/Permeabilization Concentrate and Diluent solution and Permeabilization buffer (10X) (both from Thermo Fisher Scientific, Waltham, MA, USA).

To quantify cardiac-infiltrating myeloid cells on day 3 and day 7 after MI, the cells were labeled with BV510 FVD (BD Biosciences, San Jose, CA, USA), FITC anti-CD45, PE/Cy7 anti-CD11b, APC anti-Ly6G, PE anti-F4/80, BV421 anti-Ly6C, PE anti- MHC-II, and Percp-Cy5.5 anti-CD11c (all from BioLegend, San Diego, CA, USA).

To quantify the abundance of immune cells under physiological conditions in the spleen, MLNs, and peripheral blood, the collected cells were labeled with FITC anti-CD45, PE/Cy7 anti-CD11b, APC anti-Ly6G, PE anti-CD19, APC-Cy7 anti-CD3, and Percp-Cy5.5 anti-NK1.1 (all from BioLegend, San Diego, CA, USA).

To assess the activation of T cells on day 7 after MI, cells from the heart, spleen, and MLNs were stained with BV510 FVD (BD Biosciences, San Jose, CA, USA) and FITC anti-CD45, PE/Cy7 anti-CD4, and APC anti-CD69 (all from BioLegend, San Diego, CA, USA) at 4°C in the dark. After thorough washing, the cells were fixed and permeabilized using Foxp3/Transcription Factor Fixation/Permeabilization Concentrate and Diluent solution, along with Permeabilization buffer (10X) (both from Thermo Fisher Scientific, Waltham, MA, USA), ensuring effective staining and analysis of the target antigens. Subsequently, the cells were stained with PE anti-Foxp3 and APC anti-Ki67 antibodies (eBioscience, San Diego, CA, USA).

Flow cytometry was conducted using a BD LSRFortessa X-20 instrument (BD Biosciences, San Jose, CA, USA). FlowJo (Version 10.4, TreeStar, Ashland, OR, USA) was used for data analysis.

### 2.9 RNA sequencing

Infarcted hearts from WT and *Crlf2^-/-^* mice were quickly collected on day 7 post-MI. RNA was extracted as described in Section 2.5.1, following which 1% agarose gel electrophoresis was employed to evaluate potential contamination and degradation of the samples. Quantification and qualification of RNA were performed using the RNA Nano 6000 Assay Kit (Agilent, USA) on the analyzer 2100 system (Agilent Technologies, CA, USA). mRNA was isolated from the RNA mixture using poly-T oligo-attached magnetic beads (Thermo Fisher Scientific, Waltham, MA, USA), a technique that efficiently captures and purifies mRNA molecules, ensuring their purity for downstream applications. Subsequently, cDNA synthesis was performed, and the resulting product underwent selective purification using the AMPure XP system (Beckman Coulter, Miami, FL, USA). This purification step was crucial in obtaining cDNA fragments with lengths ranging from 370 to 420 base pairs, ensuring the accuracy and reliability of downstream analysis. The quality of the library was assessed using a Qubit2.0 Fluorometer (Life technologies corporation, Gaithersburg, MD, USA), and the insert size was determined using an Agilent 2100 analyzer (Agilent Technologies, CA, USA). The Illumina NovaSeq 6000 platform (Illumina, San Diego, CA, USA) was used to sequence the library preparations, resulting in 125 bp/150 bp paired-end reads.

Processed raw data using FASTP software (v0.19.4, HaploX, Shenzhen, China) to ensure clean, high-quality data with determined Q20, Q30, and GC content for further analysis. Using Hisat2 (version 2.0.5, JHU, Baltimore, MD, USA), the reference genome was indexed to facilitate alignment. Subsequently, the paired-end clean reads were precisely mapped to the indexed reference genome. To quantify the levels of gene expression, we employed the featureCounts tool (version 1.5.0-p3). The Fragments Per Kilobase of the exon model per Million mapped reads (FPKM) for each gene was calculated, considering exon length and the number of successfully mapped reads, offering a normalized measure of gene expression, factoring in gene size and sequencing depth.

Using the DESeq2 R package (v1.20.0), we performed differential expression analysis. To enhance accuracy and reduce false positives, we adjusted the P-values using the Benjamini-Hochberg method. The threshold for significant differential expression was set at Padj ≤ 0.05 and |log2(foldchange)| ≥ 0.5. The ggplot2 R package (version 3.0.3) was used to conduct principal component analysis (PCA) and correlation analysis between WT and *Crlf2^-/-^* mice, while the VennDiagram R package was employed to generate the Venn diagram. The clusterProfiler R package (version 3.8.1) was used to perform Gene Ontology (GO) enrichment analysis on differentially expressed genes (DEGs) and to assess the statistical enrichment of genes exhibiting differential expression within Kyoto Encyclopedia of Genes and Genomes (KEGG) pathways.

### 2.10 Statistical analysis

The data are presented as mean ± standard error of the mean (SEM). Kolmogorov-Smirnov test with Lilliefors correction assessed data normality, whereas the F test evaluated variance equality. Unpaired Student’s t-test was used for normally distributed data, and Mann–Whitney U test for non-normal data. One-way ANOVA and two-way ANOVA with Tukey’s *post hoc* test analyzed multiple group differences. Kaplan–Meier method plotted survival curves, followed by the log-rank test for comparison. Significance was set at 2-tailed P < 0.05. All statistical analyses were performed using GraphPad Prism 9.0 (GraphPad Prism, San Diego, CA, USA).

## 3 Results

### 3.1 TSLP expression is elevated in the infarcted heart following MI

**Figure 1:**
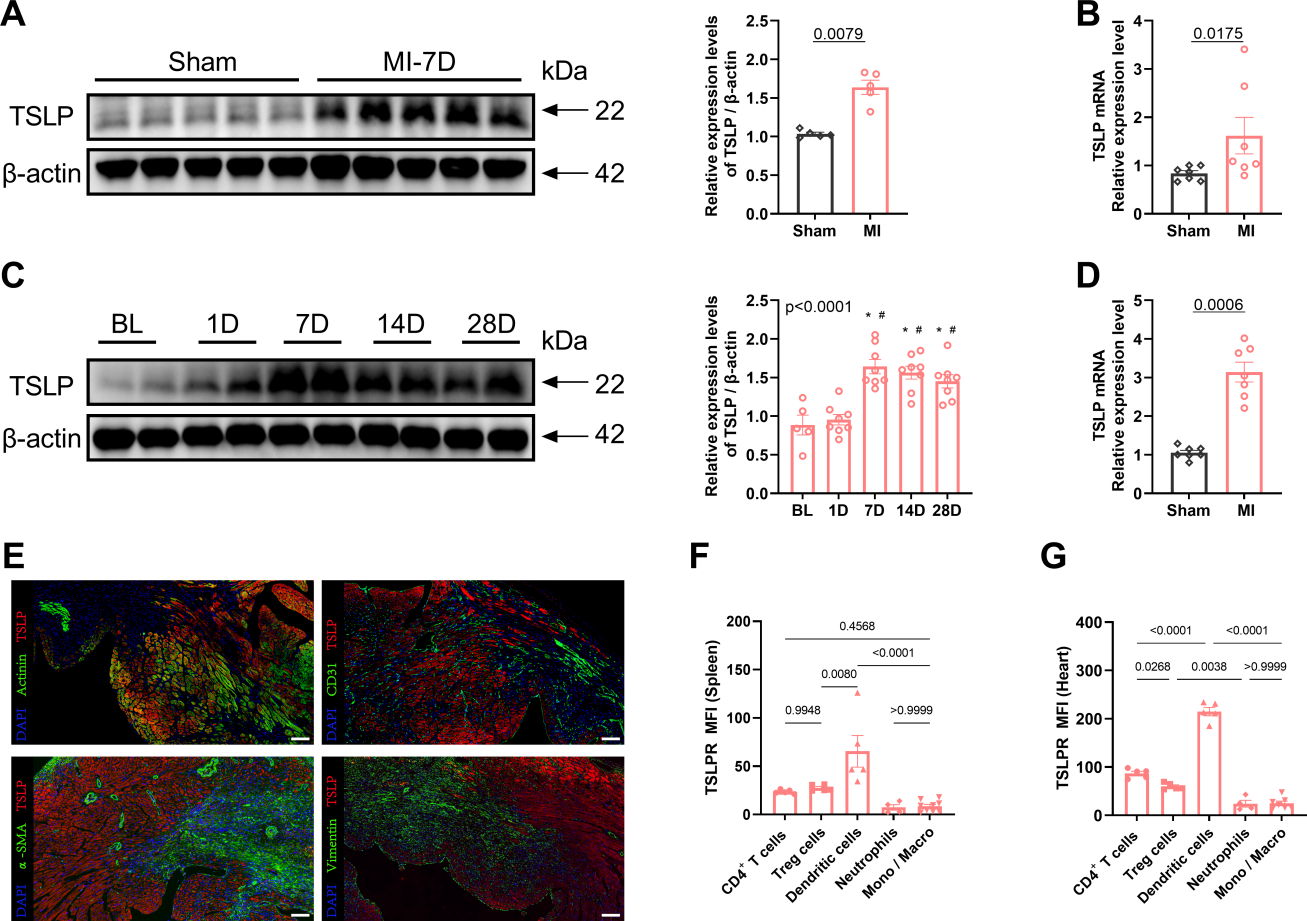
Thymic stromal lymphopoietin (TSLP) levels are elevated in cardiac tissues following myocardial infarction (MI). **(A)** MI was induced in C57/B6J mice *in vivo* and western blotting analysis of TSLP expression in heart tissues was performed on day 7 later, using β-actin as a loading control; each lane represents an independent mouse heart (n = 5 per group). **(B)** *Tlsp* mRNA levels in the heart tissues of mice on day 7 post-MI. Transcripts were normalized to glyceraldehyde-3-phosphate dehydrogenase (*Gapdh*) (n = 7 per group). **(C)** A representative western blotting analysis was conducted to assess TSLP expression in mouse cardiac tissues at baseline (BL) and day 1, day 7, day 14, and day 28 post-MI (n =5–8 per group). **(D)** *Tslp* mRNA expression was evaluated in myocardial cells isolated using the Langendorff perfusion system from mice in both the MI and sham groups on day 7 post-surgery (n = 7 per group). **(E)** TSLP representative immunofluorescence staining in myocardial tissues of mice on day 7 post-MI: TSLP (red), α-actinin (green, upper left images), CD31 (green, upper right images), alpha smooth muscle actin (α-SMA; green, bottom left images), and vimentin (green, bottom right images). The sections were counterstained with 4’,6-diamidino-2-phenylindole (DAPI) to visualize nuclei (blue; scale bar: 50 μm) (n = 5 per group). **(F, G)** Surface expression of TSLP receptor (TSLPR) on CD4^+^ T cells, T regulatory (Treg) cells, dendritic cells, neutrophils and monocytes/macrophages (Mono/Macro) in the spleen and heart on day 7 post-MI (n = 4–9 per group). Mann–Whitney U test was used to analyze the data in **(A, B, D)**, whereas the data in **(C, F, G)** were analyzed using one-way ANOVA, followed by Tukey’s multiple comparisons test. ^*^P< 0.05 compared with BL; ^#^P< 0.05 compared with 1D.

To investigate the potential involvement of TSLP in cardiac remodeling, we examined its expression in the heart. Compared with the sham-operated group, TSLP expression in the cardiac tissue of MI mice significantly increased, peaking on day 7 and gradually decreasing by day 14, yet remaining elevated until day 28 (**Figures 1A–C**, **Supplementary Figure S1**). TSLP has been reported to be highly expressed by epithelial cells, particularly in the airway epithelium (38, 39). The main parenchymal cells in cardiac tissue consist of cardiomyocytes, fibroblasts, endothelial cells, and smooth muscle cells. We observed the highest levels of TSLP expression in cardiomyocytes located in the infarct border zone on day 7 after MI (**Figures 1D, E**). Additionally, TSLPR is known to be broadly expressed in immune cells, including mast cells, myeloid lineage cells, and lymphocytes (40, 41). Therefore, we investigated TSLPR expression in various types of immune cells in infarcted hearts and spleen on day 7 after MI, then found that the highest expression of TSLPR occurred in DCs, followed by T cells (**Figures 1F, G**, **Supplementary Figure S2**).

### 3.2 The absence of TSLP/TSLPR signaling leads to reduced survival and worsened cardiac function

**Figure 2:**
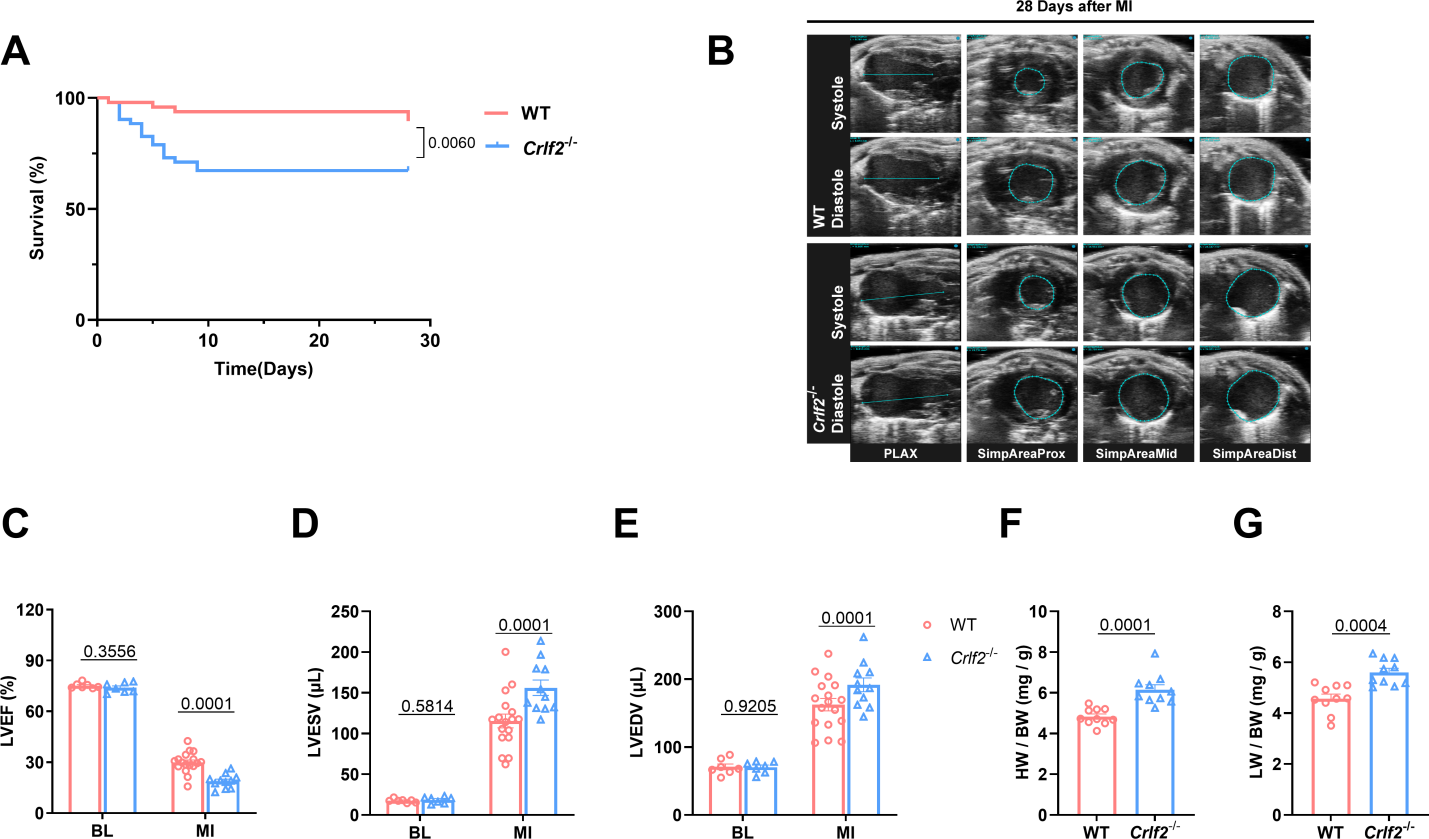
The absence of the thymic stromal lymphopoietin (TSLP)/TSLP receptor (TSLPR) signaling pathway exacerbates cardiac injury and decreases cardiac function in mice after MI. **(A)** Survival analysis of wild-type (WT)-MI mice (n = 48) and TSLPR KO (cytokine receptor like factor 2 [*Crlf2*]^-/-^)-MI mice (n = 52) up to 28 days following MI. **(B)** Representative B-mode echocardiographic images taken at 28 days post-surgery in each experimental group depicting both systolic and diastolic periods. The images include the parasternal long axis and the three parasternal short axes views from left to right. **(C–E)** Analysis of left ventricular ejection fraction (LVEF, %), left ventricular end-systolic volume (LVESV; μL), and left ventricular end-diastolic (LVEDV; μL) at baseline and on day 28 after MI (n = 7–17 per group). **(F, G)** Heart weight (HW) and lung weight (LW) were normalized to body weight (BW) after MI (n = 10 per group). Data are presented as the mean ± SEM. Survival rates were estimated using the Kaplan-Meier method and compared using log-rank tests. The data in **(C–G)** were analyzed using unpaired t-test.

To investigate the functional significance of TSLP in MI, we utilized targeted gene knockout TSLPR (*Crlf2^-/-^*) mice (**Supplementary Figure S3A**). Compared with WT mice, *Crlf2^-/-^* mice showed significantly reduced survival on day 28 post-MI (**Figure 2A**). In addition, the absence of TSLP/TSLPR signaling negatively impacted post-MI cardiac function, as evidenced by decreased LVEF and increased LVESV and LVEDV in *Crlf2^-/-^* mice compared with in WT mice on day 28 after MI (**Figures 2B–E**). Consistent with these findings, *Crlf2^-/-^* mice showed significantly higher heart weight (HW) and lung weight (LW) to body weight (BW) ratios than WT mice on day 28 after MI (**Figures 2F, G**).

### 3.3 The absence of TSLP/TSLPR signaling results in adverse ventricular remodeling

**Figure 3:**
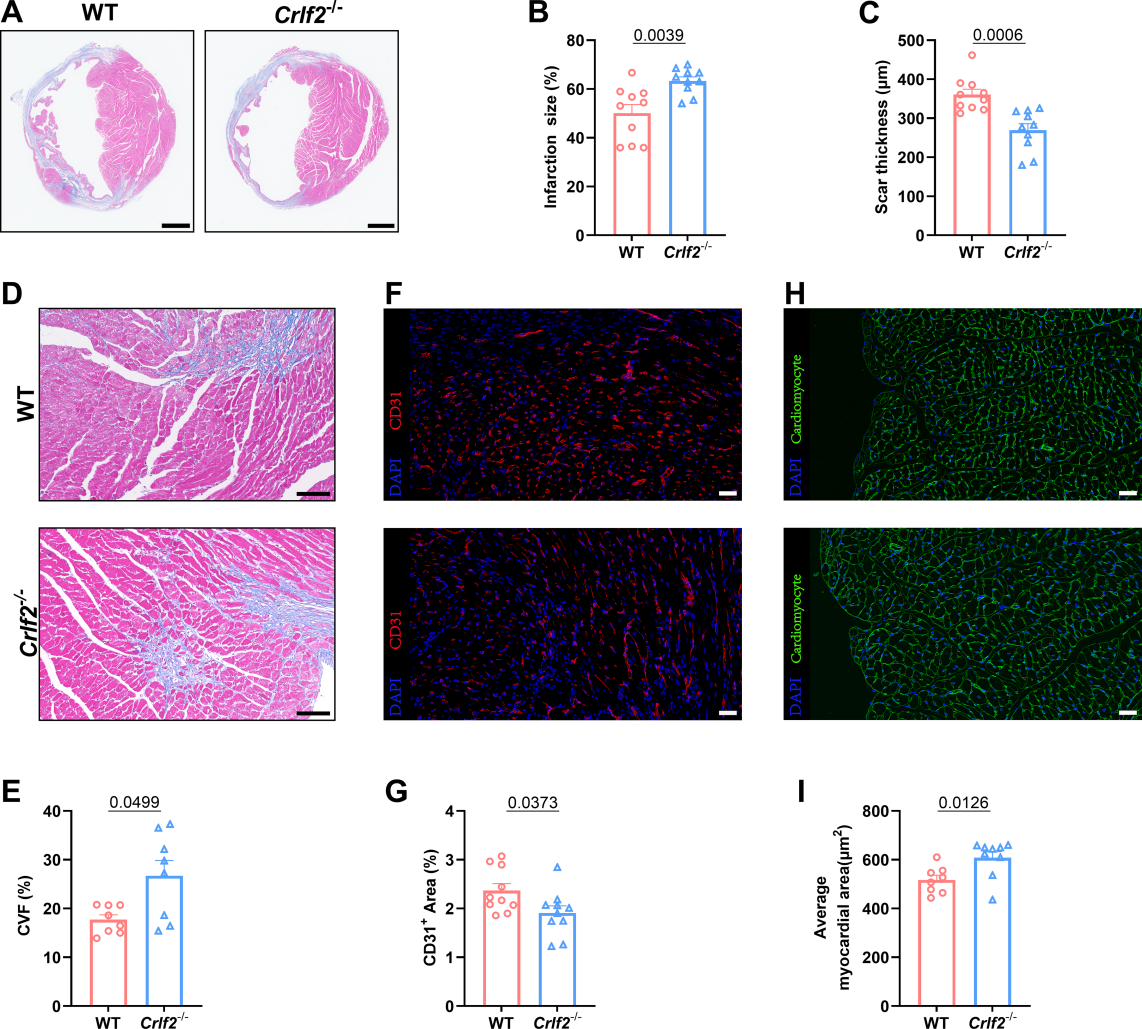
The absence of the thymic stromal lymphopoietin (TSLP)/TSLP receptor (TSLPR) signaling pathway aggravates ventricular remodeling following myocardium infarction (MI) in mice. **(A)** Heart sections were stained with Masson’s trichrome to obtain representative images (scale bar: 2 mm). **(B, C)** Quantitative analyses of infarct size and scar thickness on day 28 post-MI (n = 10 per group). **(D, E)** Representative images showing collagen volume (blue) in the border zone and quantification of the collagen volume fraction (CVF) per high-power field after Masson’s trichrome staining on day 28 post-MI (scale bar: 100 μm) (n=8 per group). **(F, G)** Representative images showing CD31^+^ staining in the peri-infarct region on day 7 post-MI (scale bar: 20 μm) and quantification of the CD31^+^ area (n = 10 per group). **(H, I)** Representative images showing WGA staining in the remote region on 28 days post- MI (scale bar: 20 μm) and quantification of the average myocardial area (n = 8–9 per group). Data are presented as the mean ± SEM. The Mann–Whitney U test was utilized to analyze the data in **(B, E)**, while unpaired t-test was employed for statistical analysis in **(C, G, I)**.

Myocardial remodeling, which refers to pathological alterations in the structure or function of the heart, has significant prognostic implications for MI (42, 43). Therefore, we investigated the potential effect of TSLP/TSLPR deficiency on cardiac repair and ventricular remodeling. Our findings indicate that, while there were no significant differences in infarct size between WT and *Crlf2^-/-^* mice at 1 day after MI, *Crlf2^-/-^* mice exhibited a larger and thinner infarct scar at 28 days post-MI (**Figures 3A–C**, **Supplementary Figures S3B, C**). Furthermore, compared with WT mice, *Crlf2^-/-^* mice exhibited increased interstitial fibrosis (**Figures 3D, E**) and reduced CD31^+^ staining (**Figures 3F, G**) in the peri-infarct region, and increased cross-sectional area of cardiomyocytes (**Figures 3H, I**) in the remote region. Nevertheless, no statistically significant difference in the proportion of TUNEL^+^ cardiomyocytes within the border area of the infarction was observed between the two groups (**Supplementary Figures S3D, E**).

### 3.4 TSLP regulates the infiltration of immune cells into the infarcted heart

**Figure 4:**
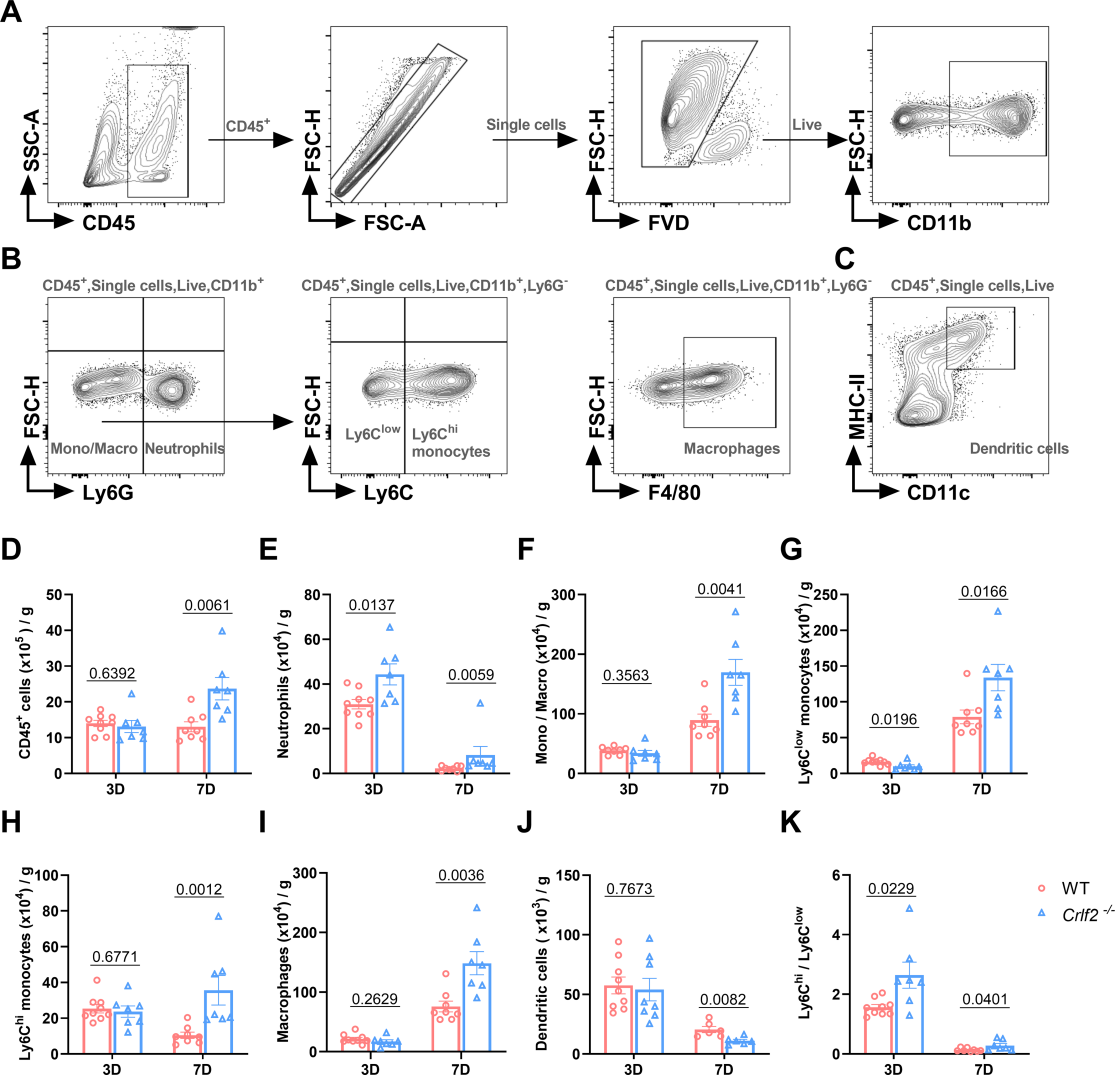
The absence of the thymic stromal lymphopoietin (TSLP)/TSLP receptor (TSLPR) signaling pathway facilitates the infiltration of myeloid cells into cardiac tissue following myocardium infarction (MI). **(A–C)** The gating strategies employed in identifying CD45^+^FVD^-^ total CD45^+^ cells, CD45^+^FVD^-^CD11b^+^Ly6G^+^ neutrophils, CD45^+^FVD^-^CD11b^+^Ly6G^-^ monocytes/macrophages (Mono/Macro), CD45^+^FVD^-^CD11b^+^Ly6G^-^Ly6C^low^ monocytes, CD45^+^FVD^-^CD11b^+^Ly6G^-^Ly6C^hi^ monocytes, CD45^+^FVD^-^CD11b^+^Ly6G^-^F4/80^+^ macrophages, and CD45^+^FVD^-^CD11c^+^MHC II^+^ dendritic cells in the heart. **(D–K)** Absolute numbers of the above cells per gram of cardiac tissue and the Ly6C^hi^/Ly6C^low^ monocyte ratio were determined on day 3 and day 7 post-MI (n = 6–9 per group). Data are presented as the mean ± SEM and unpaired t-test was used to analyze in data **(D–K)**.

**Figure 5:**
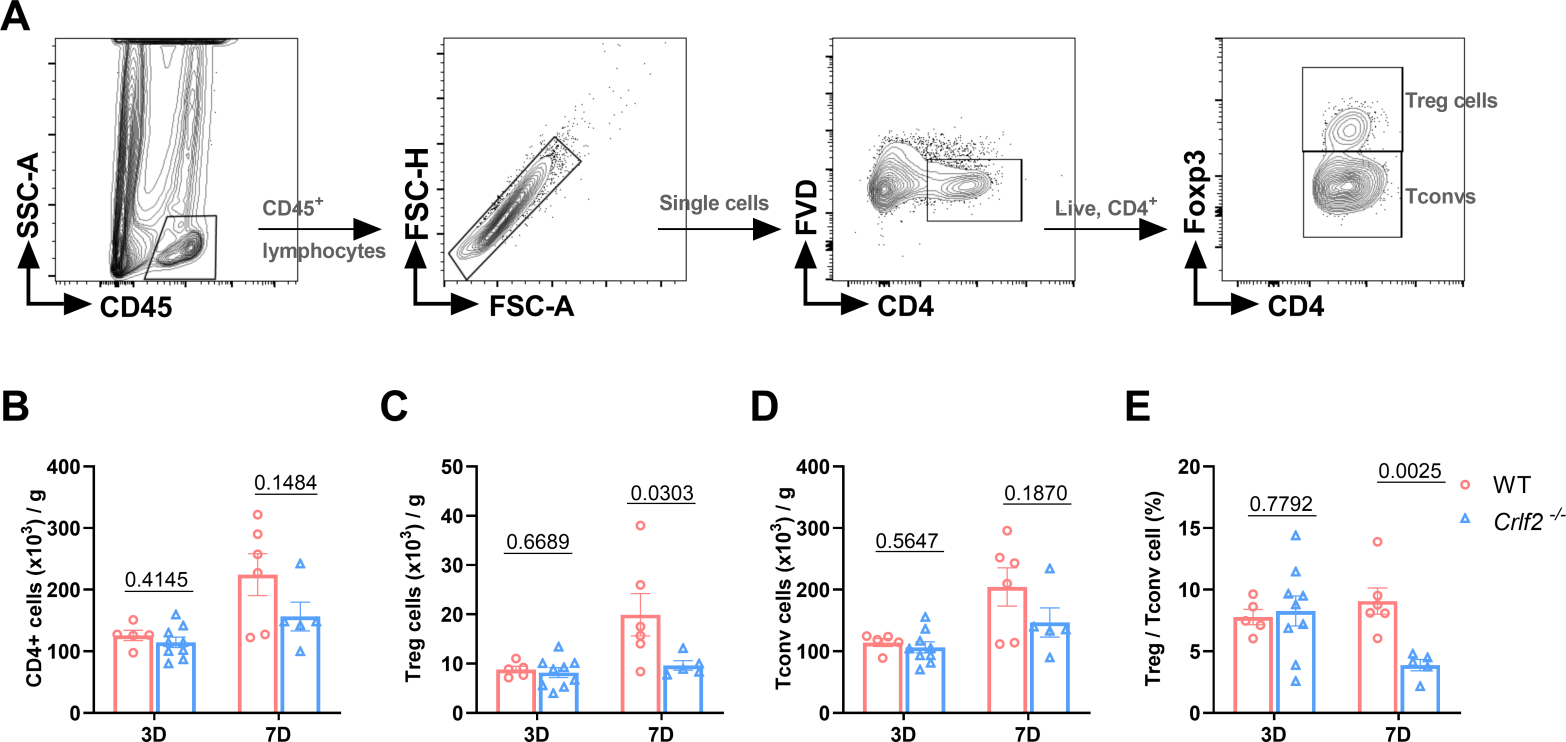
The absence of the thymic stromal lymphopoietin (TSLP)/TSLP receptor (TSLPR) signaling pathway impairs T regulatory (Treg) cells infiltration in cardiac tissue post-MI. **(A)** The gating strategies employed in identifying infiltrated CD45^+^FVD^-^CD4^+^T cells, CD45^+^FVD^-^CD4^+^Foxp3^+^ Treg cells, and CD45^+^FVD^-^CD4^+^Foxp3^-^ conventional T (Tconv) cells in the heart. **(B–E)** Absolute cell counts per gram of cardiac tissue and the Treg/Tconv cell ratio were determined on day 3 and day 7 post-MI (n = 5–9 per group). Data are presented as the mean ± SEM and were analyzed using unpaired t-test in **(B–E)**.

Previous research has established that MI triggers the infiltration of diverse innate and adaptive immune cells into cardiac tissue (44, 45). Given the pivotal role of TSLP in regulating inflammatory responses, we examined the effects of TSLPR deficiency on MI-induced inflammatory responses. We found no significant difference in the number of immune cell subsets between WT and *Crlf2^-/-^* mice in the spleen, MLNs, and blood at baseline (**Supplementary Figure S4**). After MI, *Crlf2^-/-^* mice had a significantly higher total number of heart-infiltrated CD45^+^ cells than WT mice. The gating strategies are illustrated in (**Figures 4A-C**. Infiltration of myeloid cell subsets (**Figure 5A**), including neutrophils, monocytes and macrophages, increased significantly, while DC cells decreased. Furthermore, *Crlf2^-/-^* mice showed a higher Ly6C^hi^/Ly6C^low^ monocyte ratio in the infarcted hearts (**Figures 4D–K**). In lymphocyte subsets (**Figure 5A**), a significant decrease in the number of Treg cells and a reduction in the Treg/Tconv ratio were observed post-MI in *Crlf2^-/-^* mice (**Figures 5B–E**). However, there was no alteration in the numbers of B and NK cells (**Supplementary Figures S5B, C**).

### 3.5 TSLP regulates the activation and proliferation of CD4^+^ T cells

**Figure 6:**
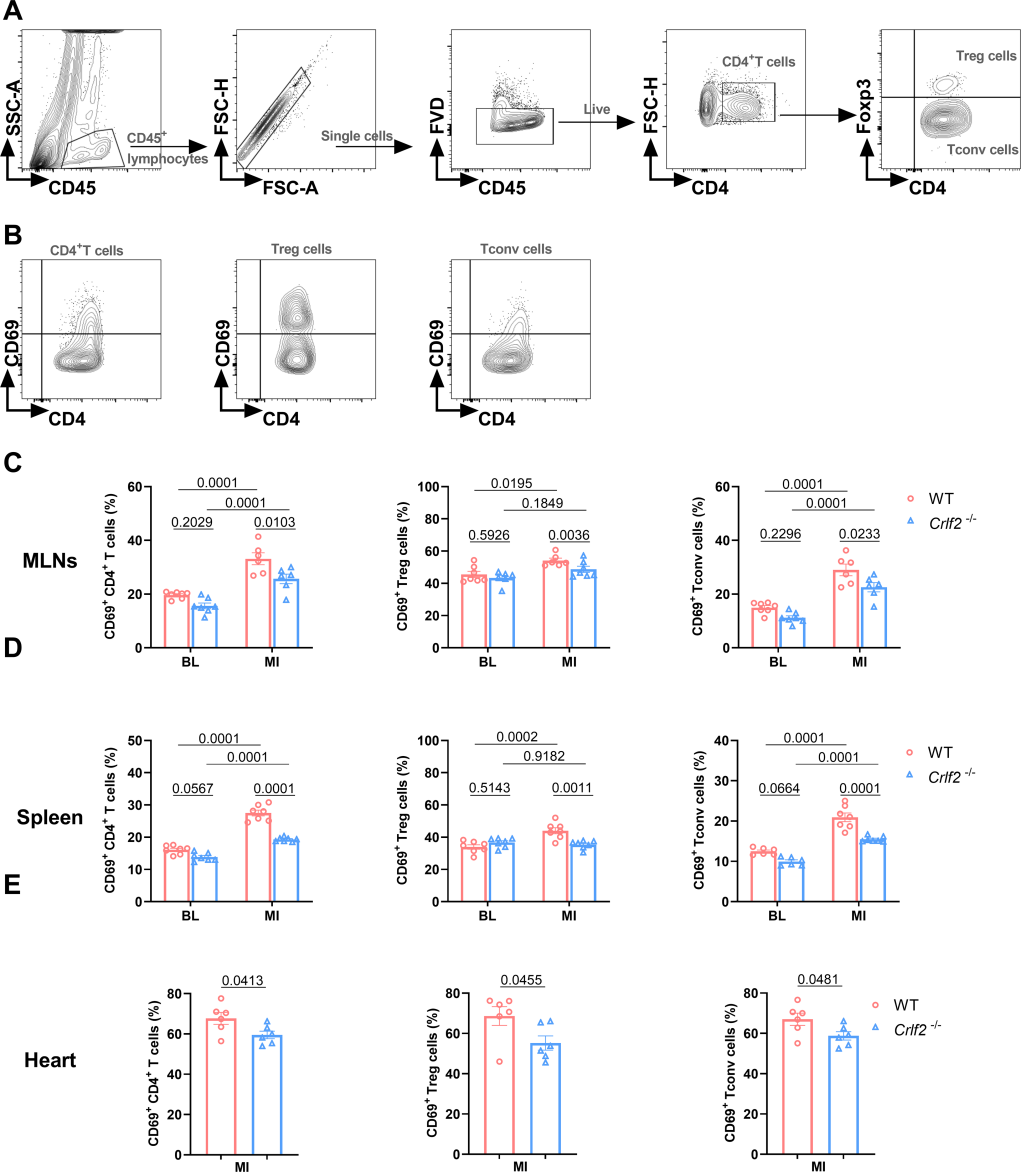
The absence of the thymic stromal lymphopoietin (TSLP)/TSLP receptor (TSLPR) signaling pathway inhibits the early activation of CD4^+^T cells and their subsets in the mediastinal lymph nodes (MLNs), spleen and heart post-MI. **(A)** The gating strategies employed in identifying CD45^+^FVD^-^CD4^+^T cells, CD45^+^FVD^-^CD4^+^Foxp3^+^ regulatory T (Treg) cells, and CD45^+^FVD^-^CD4^+^Foxp3^-^ conventional T (Tconv) cells in the heart. **(B–E)** The expression of CD69 on CD4^+^ T cells, Treg cells and Tconv cells in the MLNs and spleen at baseline (BL) and on day 7 post-MI (n = 6 per group), as well as in the heart on day 7 post-MI (n = 6–7 per group) was analyzed. Data are expressed as the mean ± SEM. Data in **(C, D)** were analyzed using two-way ANOVA, followed by Tukey’s multiple comparisons test, while unpaired t-test was used to analyze data in **(E)**.

We delved into the regulation of CD4^+^ T cells by TSLP/TSLPR signaling. CD69 is considered an early activation marker and Ki67 is considered a late activation and proliferation marker of T cells. At baseline, the absence of TSLP/TSLPR signaling had no effect on the expression of CD69 on lymphoid or splenic CD4^+^ T cells, Treg cells, and Tconv cells. After MI, the expression of CD69 on these cells increased significantly. In *Crlf2^-/-^* mice, the induction of CD69 by MI was completely abolished in Treg cells and partially abolished in CD4^+^ T cells and Tconv cells of MLNs and spleens. Furthermore, the expression of CD69 on cardiac CD4^+^ T cells, Treg cells, and Tconv cells were reduced in *Crlf2^-/-^* mice after MI (**Figure 6**).

**Figure 7:**
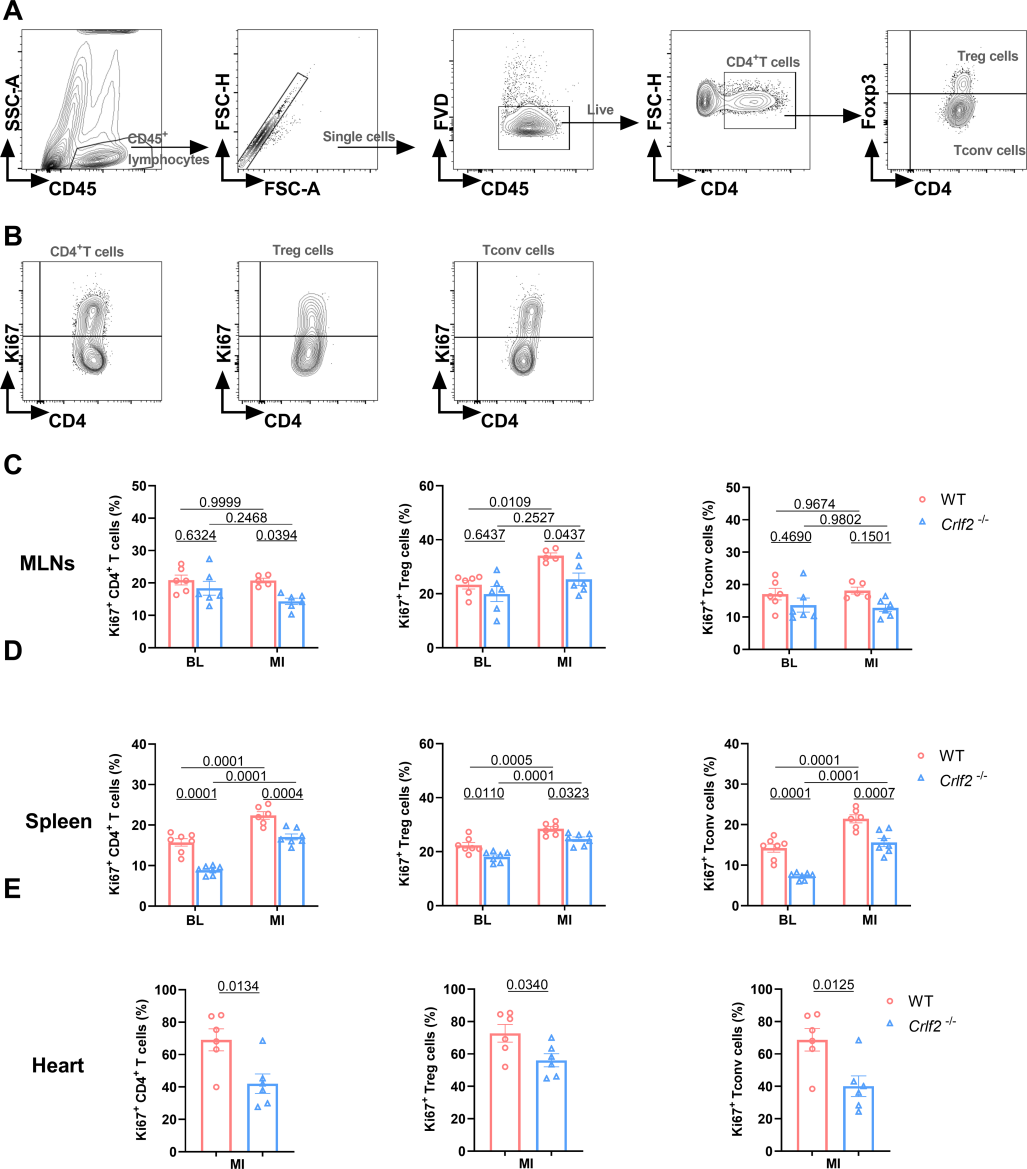
The absence of the thymic stromal lymphopoietin (TSLP)/TSLP receptor (TSLPR) signaling pathway inhibits the late activation and proliferation of CD4^+^T cells and their subsets in the mediastinal lymph nodes (MLNs), spleen and heart post-MI. **(A)** The gating strategies employed in identifying CD45^+^FVD^-^CD4^+^T cells, CD45^+^FVD^-^CD4^+^Foxp3^+^ regulatory T (Treg) cells, and CD45^+^FVD^-^CD4^+^Foxp3^-^ conventional T (Tconv) cells in the heart. **(B–E)** The expression of Ki67 in CD4^+^ T cells, Treg cells and Tconv cells in the MLNs and spleen at baseline (BL) and on day 7 post-MI (n = 6 per group), as well as in the heart on day 7 post-MI (n = 5–7 per group) was analyzed. Data are expressed as the mean ± SEM. Data in **(C, D)** were analyzed using two-way ANOVA, followed by Tukey’s multiple comparisons test, while unpaired t-test was used to analyze data in **(E)**.

At baseline, the absence of TSLP/TSLPR signaling significantly reduced the expression of Ki67 on splenic CD4^+^ T cells, Treg cells, and Tconv cells, but not on MLNs. After MI, the expression of Ki67 on splenic CD4^+^ T cells, Treg cells, and Tconv cells, as well as on lymphoid Treg cells, increased significantly. In *Crlf2^-/-^* mice, the induction of Ki67 by MI was completely abolished in lymphoid Treg cells and partially abolished in splenic CD4^+^ T cells, Treg cells and Tconv cells. Furthermore, the expression of Ki67 on cardiac CD4^+^ T cells, Treg cells, and Tconv cells was reduced in *Crlf2^-/-^* mice after MI (**Figure 7**). Taken together, TSLP regulated CD4^+^ T cells activation and proliferation at baseline and after MI, with a greater impact on Treg cells than Tconv cells.

**Figure 8:**
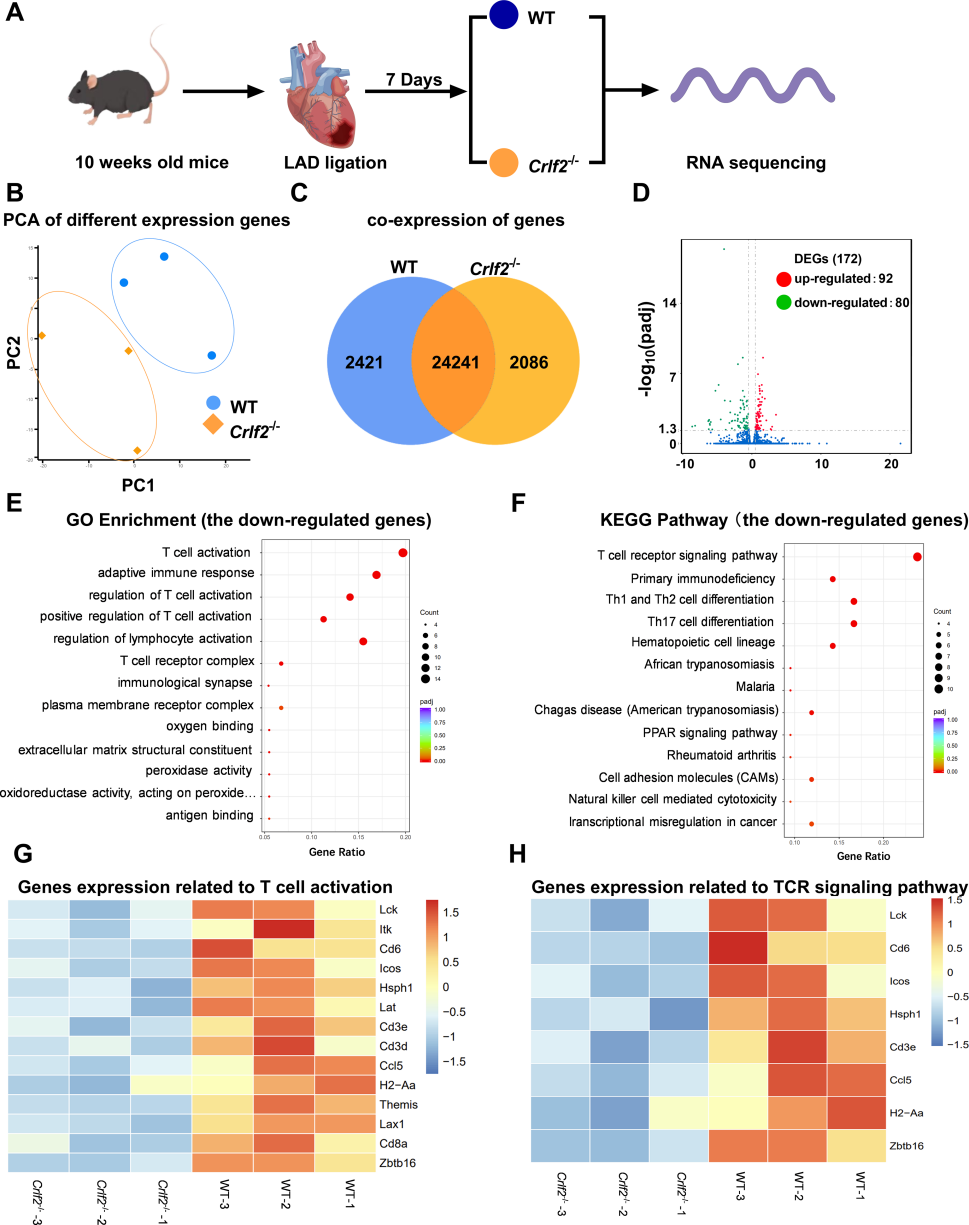
Bulk RNA sequencing was performed on heart tissues from mice subjected to left anterior descending artery (LAD) ligation. **(A)** Experimental design: on day 7 post-MI, the peri-infarcted and infarcted heart tissues were collected from wild-type (WT) and cytokine receptor like factor 2 (*Crlf2*)^-/-^ mice (n = 3 per group). **(B)** Principal component analysis (PCA) was conducted on the two groups. **(C)** Venn diagram representation of the sum of co-expressed genes in WT and *Crlf2^-/-^* mice. **(D)** A volcano plot displaying differentially expressed up- and down-regulated genes between WT and *Crlf2^-/-^* mice. **(E, F)** Gene Ontology (GO) enrichment and Kyoto Encyclopedia of Genes and Genomes (KEGG) pathway analyses of downregulated genes. **(G, H)** Heat maps illustrating the gene expression patterns associated with T-cell activation in GO enrichment and with TCR signaling pathway in KEGG pathway analysis.

To gain deeper insights, we collected the peri-infarcted and infarcted heart tissues from WT and *Crfl2^-/-^* mice on day 7 post-MI for RNA sequencing analysis (**Figure 8A**). The PCA analysis and co-expressed genes in WT and *Crlf2^-/-^* mice are presented in **Figures 8B, C**, while the correlation analysis is depicted in **Supplementary Figure S6A**. A comprehensive analysis revealed 172 DEGs with significance thresholds of an adjusted P value ≤ 0.05 and |log2(foldchange)| ≥ |0.5|. Notably, 92 of these genes were upregulated in *Crlf2^-/-^* mice compared with their WT counterparts, while 80 were downregulated, highlighting the distinct transcriptional profiles between the two groups (**Figure 8D**). GO and KEGG pathway analyses of downregulated genes revealed a reduction in T cell activation and TCR signaling in the infarcted hearts of *Crlf2^-/-^* mice (**Figures 8E, F**). Specifically, key genes involved in both T-cell activation and TCR signaling, including *Lck, Cd6, Icos, Hsph1, Cd3e, Ccl5, H2-Aa and Zbtb16*, were downregulated in *Crlf2^-/-^* mice (**Figures 8G, H**). GO and KEGG analyses of the upregulated genes in the infarcted heart of *Crlf2^-/-^* mice were presented in **Supplementary Figures S6B, C**.

**Figure 9:**
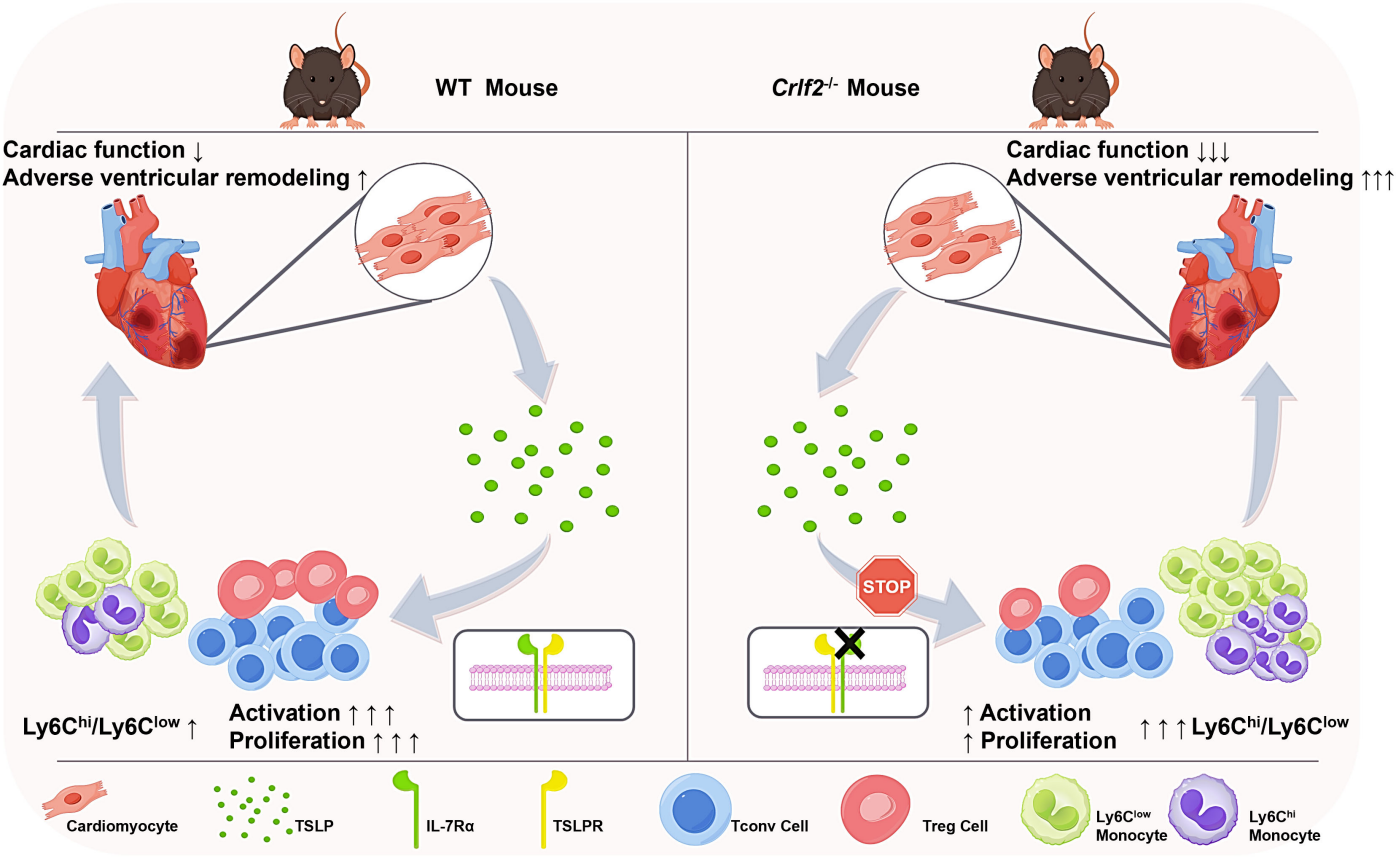
TSLP regulates immune cell response and facilitates cardiac repair after MI. TSLP expression is increased in infarcted hearts and originates primarily from cardiomyocytes in the infarct border zone. Compared to WT mice, *Crlf2* ^-/-^ mice exhibited reduced survival rates, impaired cardiac function, and increased adverse ventricular remodeling after MI. Meanwhile, the absence of TSLP-TLSPR signaling results in decreased CD4^+^ T-cell activation and proliferation, reduced Treg cell number and a lower Treg/Tconv ratio, but increased myeloid cell infiltration and a higher Ly6C^high^/Ly6C^low^ monocyte ratio.

## 4 Discussion

TSLP is a cytokine that responds to external and internal danger signals and occupies a pivotal position at the forefront of the inflammatory cascade (46). Its biological functions are pleiotropic, encompassing roles in the development, differentiation, and maintenance of immune homeostasis. In addition, TSLP contributes to the occurrence and development of various types of diseases, such as asthma, variant dermatitis, inflammatory bowel disease, and breast cancer (47–49). Despite its wide-ranging effects, the impact of TSLP on ventricular remodeling after MI has not been fully explored. In our study, we identified that TSLP was upregulated after MI. Utilizing *Crlf2^-/-^* mice, which lack TSLP/TSLPR signaling *in vivo*, we found that TSLP regulated CD4^+^ T cell activation and proliferation with a greater impact on Treg cells than on conventional T cells, thereby playing an essential role in post-MI cardiac repair.

TSLP is predominantly expressed by epithelial cells and keratinocytes (50), specifically including the epithelial cells of Hassall bodies in the human thymus (23). In pathological states, additional cell types such as mast cells, fibroblasts, dendritic cells, trophoblasts, and hepatocytes secrete TSLP (51–55). In our study, TSLP was primarily produced by myocardial cells located in the infarct border zone of mice, reaching a significant elevation on day 7 post-infarction. Recent investigations have shown that patients with acute MI exhibit higher plasma TSLP levels than those with unstable angina (29), suggesting that TSLP may trigger cardiac-specific responses in mice through autocrine or paracrine mechanisms, and systemic inflammatory responses in humans through an endocrine mechanism. Numerous effector cytokines, such as IL-1β, IL-6, and IL-10 (56) are involved in the healing process after MI, exerting direct or indirect effects. Similarly, these cytokines can induce, enhance, or inhibit TSLP production. For example, IL-1β and tumor necrosis factor-α activate nuclear factor kappa B in human airway epithelial cells, leading to TSLP production (57). Nevertheless, how MI increases TSLP secretion by myocardial cells remains unclear.

TSLP, through a heterodimer receptor consisting of TSLPR and IL-7Rα, sequentially activates intracellular Janus kinase 1/2 and signal transducer and activator of transcription 5, ultimately promoting the transcription of target genes, including those encoding IL-4/IL-13 (58). TSLPR, as a unique functional receptor of TSLP, is expressed across a range of immune cells, such as DCs, monocytes/macrophages, basophils/eosinophils, CD4^+^ T cells, CD8^+^ T cells, NK cells, and mast cells (52, 57, 59–62). Moreover, certain parenchymal cells, like human airway smooth muscle cells and tracheal fibroblasts, not only secrete TSLP but also express TSLPR, potentially amplifying the effects of TSLP (53, 63). TSLPR expression varies across different cell subpopulations and organs, with distinct expression patterns even within a single cell group depending on their resting or activated state (64). Our research revealed that after MI, TSLPR is predominantly expressed on the surface of DCs, CD4^+^T cells, and Treg cells in the myocardium. This suggests that TSLP may regulate post-infarction inflammatory responses by acting on these immune cells via paracrine or endocrine mechanisms.

Our study focused on the impact of the TSLP/TSLPR signaling pathway on the local infiltration of immune cells after MI. Consistent with previous reports (65), our findings indicated that the absence of TSLP/TSLPR signaling had no effect on the number of lymphocytes in mice at baseline levels. Following MI, neutrophils are initially recruited and reach their peak within 1-3 days; pro-inflammatory Ly6C^hi^ monocytes exhibit a peak on day 3, while, anti-inflammatory Ly6C^low^ monocytes (66), along with T and B lymphocytes show their peak on day 7 (67). Based on this observation, we investigated the impact of TSLP on immune cell infiltration in the infarcted heart on both day 3 and day 7 after MI.

Compared with WT mice, changes in the infiltration of various immune cells in the hearts of *Crlf2^-/-^* mice mainly occurred on day 7 post-MI, coinciding with a local increase in TSLP levels in the heart. In previous studies, the main immune cell subset responsive to TSLP was bone marrow-derived DCs. TSLP-activated CD11c^+^ DCs are crucial for maintaining CD4^+^ T cell homeostasis. Triggering the self-peptide MHC can induce the proliferation of immature CD4^+^ T cells, producing polyclonal T cells with a central memory phenotype and function (68). In the context of allergic diseases like asthma or atopic dermatitis, TSLP secreted by epithelial cells stimulates DCs in an antigen-specific manner, increasing the expression of surface co-stimulatory molecules such as CD80 and CD86, and inducing inflammatory helper T (Th)-2 cell polarization through OX40/OX40L interaction with immature CD4^+^ T cells, secreting cytokines such as IL-4 and IL-13. In addition, TSLP is involved in the maintenance and polarization of chronic Th2 inflammation and CRTH2^+^ Th2 effector memory cells (69, 70). DCs can also generate TSLP under certain conditions, forming a positive feedback loop (71). Some studies have also shown that during the adaptive immune process, TSLP can directly bind to TSLPR on CD4^+^ T cells in the mouse spleen and lymph nodes, thereby inducing and amplifying Th2 type inflammation (72–74).

In a series of studies, DCs have been observed to improve myocardial remodeling and cardiac function after MI by regulating the polarization of Treg cells. This type of DC possesses a certain degree of tolerance, characterized by low expression of CD40 and the costimulatory molecules CD80 and CD86 (75, 76). Meanwhile, day 7 post-MI is the peak of lymphocyte infiltration, and CD4^+^T cells consist primarily of two functionally opposing subgroups: pro-inflammatory Th1 cells that produce interferon-γ and Treg cells that support cardiac healing. Our research findings indicate a decline in the number of Treg cells on day 7 post-MI, further emphasizing the pivotal role of Treg cells in cardiac healing. Further investigation is needed to determine whether reduced DCs number is involved in the reduction of cardiac infiltrating Treg cells.

Treg cells not only facilitate cardiac healing by secreting IL-10, TGF-β, insulin-like growth factor 2, etc., but also suppress the pro-inflammatory differentiation of monocytes following MI, resulting in the downregulation of pro-inflammatory genes such as IFN-γ expression (77, 78). Previous studies have reported that in viral myocarditis, adoptive transfer of Treg cells can protect the heart from inflammatory damage and fibrosis by modulating the balance between Ly6C^high^ and Ly6C^low^ monocytes (79). Our results suggest a disruption of the balance between Ly6C^hi^ and Ly6C^low^ monocytes in *Crlf2^-/-^* mice after MI. Nevertheless, the mechanism by which TSLP mediates its effects on monocytes, either directly or indirectly via Treg cells, has not been elucidated.

According to some research reports, the activation and proliferation of CD4^+^ T cells induced by MI facilitate collagen deposition, which is crucial for preventing heart rupture and promoting wound healing (80). Nevertheless, the continuous activation of CD4^+^ T cells and excessive expansion of dysfunctional Treg cells also increase the risk of residual inflammation, aggravating adverse ventricular remodeling in chronic ischemic heart failure (81, 82). Our results suggest that TSLP/TSLPR signaling plays a significant role in the expansion and activation of CD4^+^ T cells including Treg cells and Tconv cells after MI, with a greater impact on Treg cells than Tconv cells. Consistent with these findings, RNA sequencing analysis of cardiac tissue from both groups of mice at day 7 post-MI suggested that the downregulated genes in the *Crlf2* ^-/-^ mice were primarily associated with T cell activation and the TCR signaling pathway.

In summary, our investigation underscores the cardioprotective effects of TSLP in modulating CD4^+^ T-cell response after MI, deepening our understanding of its role in cardiac repair. Our findings suggest that TSLP may play a pivotal immunomodulatory role in post-MI heart failure, hinting at its potential as a therapeutic option for patients.

## Data Availability

The datasets presented in this study can be found in online repositories. The names of the repository/repositories and accession number(s) can be found below: GSE265931 (GEO).

## Gloassary

α-SMA: Alpha smooth muscle actin
BW: Body weight
cDNA: Complementary DNA
Crlf2: Cytokine receptor life factor 2
DAPI: 4′:6-diamidino-2-phenylindole
DC: Dendritic cell
Foxp3: Forkhead box P3
FPKM: Fragments per kilobase of the exon model per million mapped fragments
GAPDH: Glyceraldehyde-3-phosphate dehydrogenase
GO: Gene Ontology
HW: Heart weight
IL: Interleukin
IL-7Rα: IL-7 receptor α
KEGG: Kyoto Encyclopedia of Genes and Genomes
LAD: Left anterior descending artery
LVEF: Left Ventricular Ejection Fraction
LVEDV: Left Ventricular End-Diastolic Volume
LVESV: Left Ventricular End Systolic Volume
LW: Lung weight
MFI: Mean fluorescence intensity
MHC: Major histocompatibility complex
MI: Myocardial infarction
MLNs: Mediastinal lymph nodes
MYHCA: Myosin heavy chain α
NK: Natural killer
PCA: Principal component analysis
SEM: Standard error of the mean
Tconv: Conventional T cell
TCR: T cell receptor
Th: Helper T cell
Treg: Regulatory T cell
TSLP: Thymic stromal lymphopoietin
TSLPR: Thymic stromal lymphopoietin receptor
TTC: 2,3,5-Triphenyl tetrazolium chloride
TUNEL: Terminal deoxynucleotidyl transferase dUTP nick-end labeling
WT: Wild-type

## References

[1] Tsao CW Aday AW Almarzooq ZI Anderson C Arora P Avery CL. Heart disease and stroke statistics-2023 update: A report from the American heart association. Circulation. (2023) 147:e93–e621. doi: 10.1161/CIR.0000000000001123 36695182 PMC12135016

[2] Townsend N Kazakiewicz D Lucy WF Timmis A Huculeci R Torbica A. Epidemiology of cardiovascular disease in Europe. Nat Rev Cardiol. (2022) 19:133–43. doi: 10.1038/s41569-021-00607-3 34497402

[3] Li S Liu Z Joseph P Hu B Yin L Tse LA. Modifiable risk factors associated with cardiovascular disease and mortality in China: a PURE substudy. Eur Heart J. (2022) 43:2852–63. doi: 10.1093/eurheartj/ehac268 35731140

[4] Bhatt DL Lopes RD Harrington RA. Diagnosis and treatment of acute coronary syndromes: A review. JAMA. (2022) 327:662–75. doi: 10.1001/jama.2022.0358 35166796

[5] Christensen DM Schjerning AM Smedegaard L Charlot MG Ravn PB Ruwald AC. Long-term mortality, cardiovascular events, and bleeding in stable patients 1 year after myocardial infarction: a Danish nationwide study. Eur Heart J. (2023) 44:488–98. doi: 10.1093/eurheartj/ehac667 PMC9902154 36433809

[6] Verma S Fedak PW Weisel RD Butany J Rao V Maitland A. Fundamentals of reperfusion injury for the clinical cardiologist. Circulation. (2002) 105:2332–6. doi: 10.1161/01.cir.0000016602.96363.36 12021216

[7] Yusuf S Joseph P Rangarajan S Islam S Mente A Hystad P. Modifiable risk factors, cardiovascular disease, and mortality in 155 722 individuals from 21 high-income, middle-income, and low-income countries (PURE): a prospective cohort study. Lancet. (2020) 395:795–808. doi: 10.1016/S0140-6736(19)32008-2 31492503 PMC8006904

[8] Vogel B Claessen BE Arnold SV Chan D Cohen DJ Giannitsis E. ST-segment elevation myocardial infarction. Nat Rev Dis Primers. (2019) 5:39. doi: 10.1038/s41572-019-0090-3 31171787

[9] Gerber Y Weston SA Enriquez-Sarano M Manemann SM Chamberlain AM Jiang R. Atherosclerotic burden and heart failure after myocardial infarction. JAMA Cardiol. (2016) 1:156–62. doi: 10.1001/jamacardio.2016.0074 PMC4957651 27437886

[10] Velagaleti RS Pencina MJ Murabito JM Wang TJ Parikh NI D'Agostino RB. Long-term trends in the incidence of heart failure after myocardial infarction. Circulation. (2008) 118:2057–62. doi: 10.1161/CIRCULATIONAHA.108.784215 PMC2729712 18955667

[11] Soumelis V Reche PA Kanzler H Yuan W Edward G Homey B. Human epithelial cells trigger dendritic cell mediated allergic inflammation by producing TSLP. Nat Immunol. (2002) 3:673–80. doi: 10.1038/ni805 12055625

[12] Pandey A Ozaki K Baumann H Levin SD Puel A Farr AG. Cloning of a receptor subunit required for signaling by thymic stromal lymphopoietin. Nat Immunol. (2000) 1:59–64. doi: 10.1038/76923 10881176

[13] Corren J Ziegler SF. TSLP: from allergy to cancer. Nat Immunol. (2019) 20:1603–9. doi: 10.1038/s41590-019-0524-9 31745338

[14] Friend SL Hosier S Nelson A Foxworthe D Williams DE Farr A. A thymic stromal cell line supports in *vitro* development of surface IgM+ B cells and produces a novel growth factor affecting B and T lineage cells. Exp Hematol. (1994) 22:321–8. 8112430

[15] Verstraete K Peelman F Braun H Lopez J Van Rompaey D Dansercoer A. Structure and antagonism of the receptor complex mediated by human TSLP in allergy and asthma. Nat Commun. (2017) 8:14937. doi: 10.1038/ncomms14937 28368013 PMC5382266

[16] West EE Spolski R Kazemian M Yu ZX Kemper C Leonard WJ. A TSLP-complement axis mediates neutrophil killing of methicillin-resistant Staphylococcus aureus. Sci Immunol. (2016) 1. doi: 10.1126/sciimmunol.aaf8471 PMC8530006 28783679

[17] Roberts KG Li Y Payne-Turner D Harvey RC Yang YL Pei D. Targetable kinase-activating lesions in Ph-like acute lymphoblastic leukemia. N Engl J Med. (2014) 371:1005–15. doi: 10.1056/NEJMoa1403088 PMC4191900 25207766

[18] Choa R Tohyama J Wada S Meng H Hu J Okumura M. Thymic stromal lymphopoietin induces adipose loss through sebum hypersecretion. Science. (2021) 373. doi: 10.1126/science.abd2893 PMC8917823 34326208

[19] Tahaghoghi-Hajghorbani S Ajami A Ghorbanalipoor S Hosseini-Khah Z Taghiloo S Khaje-Enayati P. Protective effect of TSLP and IL-33 cytokines in ulcerative colitis. Auto Immun Highlights. (2019) 10:1. doi: 10.1186/s13317-019-0110-z 30868311 PMC6416230

[20] Ito T Wang YH Duramad O Hori T Delespesse GJ Watanabe N. TSLP-activated dendritic cells induce an inflammatory T helper type 2 cell response through OX40 ligand. J Exp Med. (2005) 202:1213–23. doi: 10.1084/jem.20051135 PMC2213234 16275760

[21] Kim BS Siracusa MC Saenz SA Noti M Monticelli LA Sonnenberg GF. TSLP elicits IL-33-independent innate lymphoid cell responses to promote skin inflammation. Sci Transl Med. (2013) 5:170ra16. doi: 10.1126/scitranslmed.3005374 PMC3637661 23363980

[22] Hoy SM. Tezepelumab: first approval. Drugs. (2022) 82:461–8. doi: 10.1007/s40265-022-01679-2 35184265

[23] Wang Y Watanabe N Liu Y Lee HK Cao W Wang Y. Hassall's corpuscles instruct dendritic cells to induce CD4 + CD25 + regulatory T cells in human thymus. Nature. (2005) 436:1181–5. doi: 10.1038/nature03886 16121185

[24] Leichner TM Satake A Harrison VS Tanaka Y Archambault AS Kim BS. Skin-derived TSLP systemically expands regulatory T cells. J Autoimmun. (2017) 79:39–52. doi: 10.1016/j.jaut.2017.01.003 28126203 PMC5386815

[25] Kashiwagi M Hosoi J Lai JF Brissette J Ziegler SF Morgan BA. Direct control of regulatory T cells by keratinocytes. Nat Immunol. (2017) 18:334–43. doi: 10.1038/ni.3661 PMC5310986 28092372

[26] Frangogiannis NG. The inflammatory response in myocardial injury, repair, and remodelling. Nat Rev Cardiol. (2014) 11:255–65. doi: 10.1038/nrcardio.2014.28 PMC4407144 24663091

[27] Abbate A Toldo S Marchetti C Kron J Van Tassell BW Dinarello CA. Interleukin-1 and the inflammasome as therapeutic targets in cardiovascular disease. Circ Res. (2020) 126:1260–80. doi: 10.1161/CIRCRESAHA.120.315937 PMC8760628 32324502

[28] Prabhu SD Frangogiannis NG. The biological basis for cardiac repair after myocardial infarction: from inflammation to fibrosis. Circ Res. (2016) 119:91–112. doi: 10.1161/CIRCRESAHA.116.303577 27340270 PMC4922528

[29] Zhao Y Zhang Y Guo Z Ma Z Liu Y Han C. Elevated plasma thymic stromal lymphopoietin after acute myocardial infarction. Front Cardiovasc Med. (2022) 9:685677. doi: 10.3389/fcvm.2022.685677 35321112 PMC8936131

[30] Gao E Lei YH Shang X Huang ZM Zuo L Boucher M. A novel and efficient model of coronary artery ligation and myocardial infarction in the mouse. Circ Res. (2010) 107:1445–53. doi: 10.1161/CIRCRESAHA.110.223925 PMC3005817 20966393

[31] Xia N Lu Y Gu M Li N Liu M Jiao J. A unique population of regulatory T cells in heart potentiates cardiac protection from myocardial infarction. Circulation. (2020) 142:1956–73. doi: 10.1161/CIRCULATIONAHA.120.046789 32985264

[32] Bell RM Mocanu MM Yellon DM. Retrograde heart perfusion: the Langendorff technique of isolated heart perfusion. J Mol Cell Cardiol. (2011) 50:940–50. doi: 10.1016/j.yjmcc.2011.02.018 21385587

[33] Takagawa J Zhang Y Wong ML Sievers RE Kapasi NK Wang Y. Myocardial infarct size measurement in the mouse chronic infarction model: comparison of area- and length-based approaches. J Appl Physiol (1985). (2007) . 102:2104–11. doi: 10.1152/japplphysiol.00033.2007 PMC2675697 17347379

[34] Jiao J He S Wang Y Lu Y Gu M Li D. Regulatory B cells improve ventricular remodeling after myocardial infarction by modulating monocyte migration. Basic Res Cardiol. (2021) 116:46. doi: 10.1007/s00395-021-00886-4 34302556 PMC8310480

[35] Tang TT Li YY Li JJ Wang K Han Y Dong WY. Liver-heart crosstalk controls IL-22 activity in cardiac protection after myocardial infarction. Theranostics. (2018) 8:4552–62. doi: 10.7150/thno.24723 PMC6134935 30214638

[36] Tang X Wang P Zhang R Watanabe I Chang E Vinayachandran V. KLF2 regulates neutrophil activation and thrombosis in cardiac hypertrophy and heart failure progression. J Clin Invest. (2022) 132. doi: 10.1172/JCI147191 PMC8803339 34793333

[37] Heinen A Raupach A Behmenburg F Holscher N Flogel U Kelm M. Echocardiographic analysis of cardiac function after infarction in mice: validation of single-plane long-axis view measurements and the Bi-Plane Simpson method. Ultrasound Med Biol. (2018) 44:1544–55. doi: 10.1016/j.ultrasmedbio.2018.03.020 29706407

[38] Hammad H Lambrecht BN. Barrier epithelial cells and the control of type 2 immunity. Immunity. (2015) 43:29–40. doi: 10.1016/j.immuni.2015.07.007 26200011

[39] Hirose K Iwata A Tamachi T Nakajima H. Allergic airway inflammation: key players beyond the Th2 cell pathway. Immunol Rev. (2017) 278:145–61. doi: 10.1111/imr.12540 28658544

[40] Roan F Bell BD Stoklasek TA Kitajima M Han H Ziegler SF. The multiple facets of thymic stromal lymphopoietin (TSLP) during allergic inflammation and beyond. J Leukoc Biol. (2012) 91:877–86. doi: 10.1189/jlb.1211622 PMC3360473 22442496

[41] Roan F Obata-Ninomiya K Ziegler SF. Epithelial cell-derived cytokines: more than just signaling the alarm. J Clin Invest. (2019) 129:1441–51. doi: 10.1172/JCI124606 PMC6436879 30932910

[42] Frangogiannis NG. The extracellular matrix in myocardial injury, repair, and remodeling. J Clin Invest. (2017) 127:1600–12. doi: 10.1172/JCI87491 PMC5409799 28459429

[43] Frantz S Hundertmark MJ Schulz-Menger J Bengel FM Bauersachs J. Left ventricular remodelling post-myocardial infarction: pathophysiology, imaging, and novel therapies. Eur Heart J. (2022) 43:2549–61. doi: 10.1093/eurheartj/ehac223 PMC9336586 35511857

[44] Silvestre-Roig C Braster Q Ortega-Gomez A Soehnlein O. Neutrophils as regulators of cardiovascular inflammation. Nat Rev Cardiol. (2020) 17:327–40. doi: 10.1038/s41569-019-0326-7 31996800

[45] Lawler PR Bhatt DL Godoy LC Luscher TF Bonow RO Verma S. Targeting cardiovascular inflammation: next steps in clinical translation. Eur Heart J. (2021) 42:113–31. doi: 10.1093/eurheartj/ehaa099 32176778

[46] Gauvreau GM Bergeron C Boulet LP Cockcroft DW Cote A Davis BE. Sounding the alarmins-The role of alarmin cytokines in asthma. Allergy. (2023) 78:402–17. doi: 10.1111/all.15609 PMC10108333 36463491

[47] Matera MG Rogliani P Calzetta L Cazzola M. TSLP inhibitors for asthma: current status and future prospects. Drugs. (2020) 80:449–58. doi: 10.1007/s40265-020-01273-4 32078149

[48] Park JH Jeong DY Peyrin-Biroulet L Eisenhut M Shin JI. Insight into the role of TSLP in inflammatory bowel diseases. Autoimmun Rev. (2017) 16:55–63. doi: 10.1016/j.autrev.2016.09.014 27697608

[49] Stanbery AG Shuchi S Jakob VM Tait WE Ziegler SF. TSLP, IL-33, and IL-25: Not just for allergy and helminth infection. J Allergy Clin Immunol. (2022) 150:1302–13. doi: 10.1016/j.jaci.2022.07.003 PMC9742339 35863509

[50] Sims JE Williams DE Morrissey PJ Garka K Foxworthe D Price V. Molecular cloning and biological characterization of a novel murine lymphoid growth factor. J Exp Med. (2000) 192:671–80. doi: 10.1084/jem.192.5.671 PMC2193273 10974033

[51] Nagarkar DR Poposki JA Comeau MR Biyasheva A Avila PC Schleimer RP. Airway epithelial cells activate TH2 cytokine production in mast cells through IL-1 and thymic stromal lymphopoietin. J Allergy Clin Immunol. (2012) 130:225–32.e4. doi: 10.1016/j.jaci.2012.04.019 22633328 PMC3387295

[52] Guo PF Du MR Wu HX Lin Y Jin LP Li DJ. Thymic stromal lymphopoietin from trophoblasts induces dendritic cell-mediated regulatory TH2 bias in the decidua during early gestation in humans. Blood. (2010) 116:2061–9. doi: 10.1182/blood-2009-11-252940 20538796

[53] Smelter DF Sathish V Thompson MA Pabelick CM Vassallo R Prakash YS. Thymic stromal lymphopoietin in cigarette smoke-exposed human airway smooth muscle. J Immunol. (2010) 185:3035–40. doi: 10.4049/jimmunol.1000252 PMC3681514 20660708

[54] Kashyap M Rochman Y Spolski R Samsel L Leonard WJ. Thymic stromal lymphopoietin is produced by dendritic cells. J Immunol. (2011) 187:1207–11. doi: 10.4049/jimmunol.1100355 PMC3140600 21690322

[55] Li S Yi Z Deng M Scott MJ Yang C Li W. TSLP protects against liver I/R injury via activation of the PI3K/Akt pathway. JCI Insight. (2019) 4:e129013. doi: 10.1172/jci.insight.129013 31723054 PMC6948857

[56] Matter MA Paneni F Libby P Frantz S Stahli BE Templin C. Inflammation in acute myocardial infarction: the good, the bad and the ugly. Eur Heart J. (2024) 45:89–103. doi: 10.1093/eurheartj/ehad486 37587550 PMC10771378

[57] Allakhverdi Z Comeau MR Jessup HK Yoon BR Brewer A Chartier S. Thymic stromal lymphopoietin is released by human epithelial cells in response to microbes, trauma, or inflammation and potently activates mast cells. J Exp Med. (2007) 204:253–8. doi: 10.1084/jem.20062211 PMC2118732 17242164

[58] Ebina-Shibuya R Leonard WJ. Role of thymic stromal lymphopoietin in allergy and beyond. Nat Rev Immunol. (2023) 23:24–37. doi: 10.1038/s41577-022-00735-y 35650271 PMC9157039

[59] Zhou B Comeau MR De Smedt T Liggitt HD Dahl ME Lewis DB. Thymic stromal lymphopoietin as a key initiator of allergic airway inflammation in mice. Nat Immunol. (2005) 6:1047–53. doi: 10.1038/ni1247 16142237

[60] Wong CK Hu S Cheung PF Lam CW. Thymic stromal lymphopoietin induces chemotactic and prosurvival effects in eosinophils: implications in allergic inflammation. Am J Respir Cell Mol Biol. (2010) 43:305–15. doi: 10.1165/rcmb.2009-0168OC 19843704

[61] Rochman Y Leonard WJ. The role of thymic stromal lymphopoietin in CD8+ T cell homeostasis. J Immunol. (2008) 181:7699–705. doi: 10.4049/jimmunol.181.11.7699 PMC2735224 19017958

[62] Tatsuno K Fujiyama T Yamaguchi H Waki M Tokura Y. TSLP directly interacts with skin-homing Th2 cells highly expressing its receptor to enhance IL-4 production in atopic dermatitis. J Invest Dermatol. (2015) 135:3017–24. doi: 10.1038/jid.2015.318 26288354

[63] Semlali A Jacques E Koussih L Gounni AS Chakir J. Thymic stromal lymphopoietin-induced human asthmatic airway epithelial cell proliferation through an IL-13-dependent pathway. J Allergy Clin Immunol. (2010) 125:844–50. doi: 10.1016/j.jaci.2010.01.044 20236697

[64] Domeier PP Rahman Z Ziegler SF. B cell- and T cell-intrinsic regulation of germinal centers by thymic stromal lymphopoietin signaling. Sci Immunol. (2023) 8:eadd9413. doi: 10.1126/sciimmunol.add9413 36608149 PMC10162646

[65] Al-Shami A Spolski R Kelly J Fry T Schwartzberg PL Pandey A. A role for thymic stromal lymphopoietin in CD4(+) T cell development. J Exp Med. (2004) 200:159–68. doi: 10.1084/jem.20031975 PMC2212020 15263024

[66] Ong SB Hernandez-Resendiz S Crespo-Avilan GE Mukhametshina RT Kwek XY Cabrera-Fuentes HA. Inflammation following acute myocardial infarction: Multiple players, dynamic roles, and novel therapeutic opportunities. Pharmacol Ther. (2018) 186:73–87. doi: 10.1016/j.pharmthera.2018.01.001 29330085 PMC5981007

[67] Zhang R Cochran BJ Thomas SR Rye KA. Impact of reperfusion on temporal immune cell dynamics after myocardial infarction. J Am Heart Assoc. (2023) 12:e027600. doi: 10.1161/JAHA.122.027600 36789837 PMC10111498

[68] Watanabe N Hanabuchi S Soumelis V Yuan W Ho S de Waal MR. Human thymic stromal lymphopoietin promotes dendritic cell-mediated CD4+ T cell homeostatic expansion. Nat Immunol. (2004) 5:426–34. doi: 10.1038/ni1048 14991051

[69] Ito T Liu YJ Arima K. Cellular and molecular mechanisms of TSLP function in human allergic disorders–TSLP programs the "Th2 code" in dendritic cells. Allergol Int. (2012) 61:35–43. doi: 10.2332/allergolint.11-RAI-0376 22189594 PMC3660852

[70] Wang YH Ito T Wang YH Homey B Watanabe N Martin R. Maintenance and polarization of human TH2 central memory T cells by thymic stromal lymphopoietin-activated dendritic cells. Immunity. (2006) 24:827–38. doi: 10.1016/j.immuni.2006.03.019 16782037

[71] Han F Guo H Wang L Zhang Y Sun L Dai C. TSLP produced by aspergillus fumigatus-stimulated DCs promotes a Th17 response through the JAK/STAT signaling pathway in fungal keratitis. Invest Ophthalmol Vis Sci. (2020) 61:24. doi: 10.1167/iovs.61.14.24 PMC7757613 33346778

[72] Ochiai S Jagot F Kyle RL Hyde E White RF Prout M. Thymic stromal lymphopoietin drives the development of IL-13(+) Th2 cells. Proc Natl Acad Sci U.S.A. (2018) 115:1033–8. doi: 10.1073/pnas.1714348115 PMC5798347 29339496

[73] Kabata H Flamar AL Mahlakoiv T Moriyama S Rodewald HR Ziegler SF. Targeted deletion of the TSLP receptor reveals cellular mechanisms that promote type 2 airway inflammation. Mucosal Immunol. (2020) 13:626–36. doi: 10.1038/s41385-020-0266-x PMC7311324 32066836

[74] He R Oyoshi MK Garibyan L Kumar L Ziegler SF Geha RS. TSLP acts on infiltrating effector T cells to drive allergic skin inflammation. Proc Natl Acad Sci U.S.A. (2008) 105:11875–80. doi: 10.1073/pnas.0801532105 PMC2575291 18711124

[75] Choo EH Lee JH Park EH Park HE Jung NC Kim TH. Infarcted myocardium-primed dendritic cells improve remodeling and cardiac function after myocardial infarction by modulating the regulatory T cell and macrophage polarization. Circulation. (2017) 135:1444–57. doi: 10.1161/CIRCULATIONAHA.116.023106 28174192

[76] Zhu R Sun H Yu K Zhong Y Shi H Wei Y. Interleukin-37 and dendritic cells treated with interleukin-37 plus troponin I ameliorate cardiac remodeling after myocardial infarction. J Am Heart Assoc. (2016) 5:e004606. doi: 10.1161/JAHA.116.004406 PMC5210436 27919929

[77] Gladow N Hollmann C Weirather J Ding X Burkard M Uehlein S. Role of CD4(+) T-cells for regulating splenic myelopoiesis and monocyte differentiation after experimental myocardial infarction. Basic Res Cardiol. (2024) 119:261–75. doi: 10.1007/s00395-024-01035-3 PMC11008073 38436707

[78] Wang Y Dembowsky K Chevalier E Stuve P Korf-Klingebiel M Lochner M. C-X-C motif chemokine receptor 4 blockade promotes tissue repair after myocardial infarction by enhancing regulatory T cell mobilization and immune-regulatory function. Circulation. (2019) 139:1798–812. doi: 10.1161/CIRCULATIONAHA.118.036053 PMC6467561 30696265

[79] Pappritz K Savvatis K Miteva K Kerim B Dong F Fechner H. Immunomodulation by adoptive regulatory T-cell transfer improves Coxsackievirus B3-induced myocarditis. FASEB J. (2018) 32, 6066–78. doi: 10.1096/fj.201701408R 29863913

[80] Hofmann U Beyersdorf N Weirather J Podolskaya A Bauersachs J Ertl G. Activation of CD4+ T lymphocytes improves wound healing and survival after experimental myocardial infarction in mice. Circulation. (2012) 125:1652–63. doi: 10.1161/CIRCULATIONAHA.111.044164 22388323

[81] Bansal SS Ismahil MA Goel M Patel B Hamid T Rokosh G. Activated T lymphocytes are essential drivers of pathological remodeling in ischemic heart failure. Circ Heart Fail. (2017) 10:e003688. doi: 10.1161/CIRCHEARTFAILURE.116.003688 28242779 PMC5331621

[82] Bansal SS Ismahil MA Goel M Zhou G Rokosh G Hamid T. Dysfunctional and proinflammatory regulatory T-lymphocytes are essential for adverse cardiac remodeling in ischemic cardiomyopathy. Circulation. (2019) 139:206–21. doi: 10.1161/CIRCULATIONAHA.118.036065 PMC6322956 30586716

