# Supplementary material for: Thymic stromal lymphopoietin modulates T cell response and improves cardiac repair post-myocardial infarction
Source: Front Immunol. 2024 Dec 5;15:1467095. doi: 10.3389/fimmu.2024.1467095 (PMC11655303; doi:10.3389/fimmu.2024.1467095)
Supplement: Supplementary file 1 [file DataSheet1.pdf]

Supplementary Material

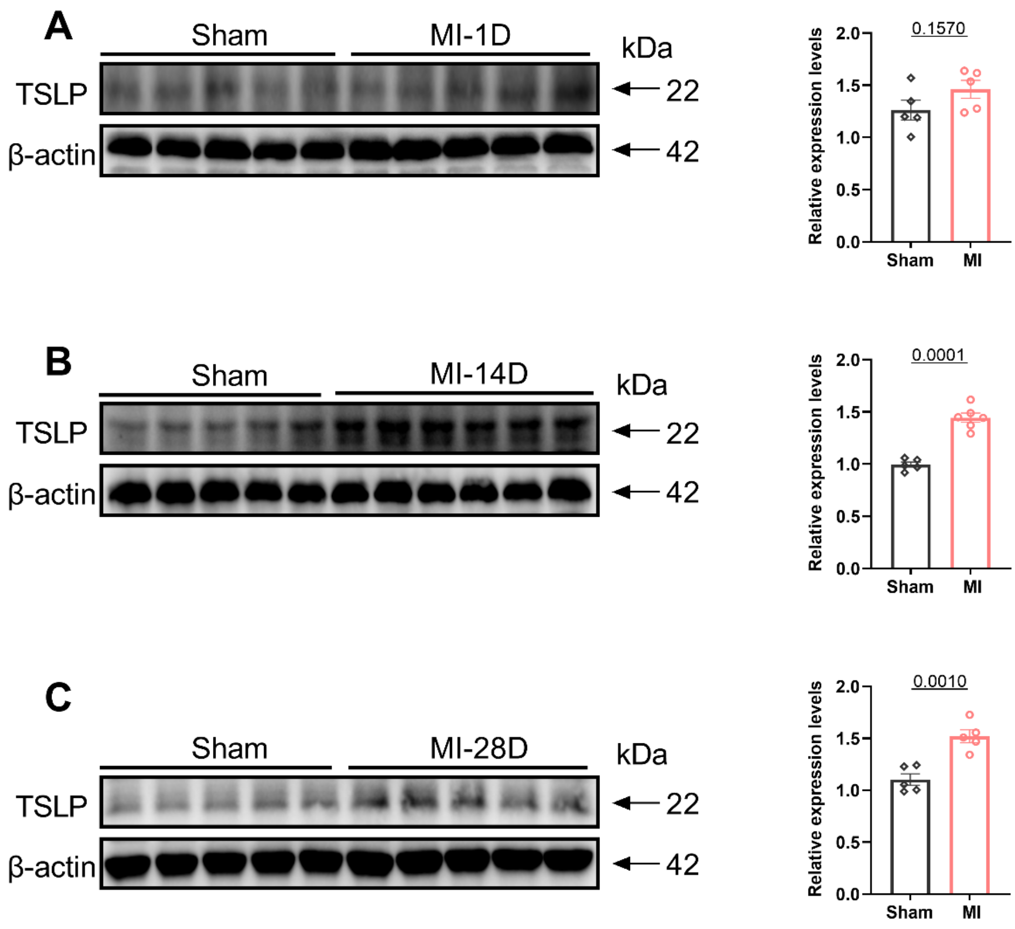

Supplementary Figure 1. Thymic stromal lymphopoietin (TSLP) expression in mouse cardiac tissue at various times. (A–C) Western blotting of TSLP level and quantitative analysis in heart tissues on day 1, day 14, and day 28 post-infarction, using β-actin as a loading control. Each lane corresponds to a sample of a mouse heart (n = 5–6 per group). Data are presented as the mean ± SEM. Data in A–C were analyzed using unpaired t-test.

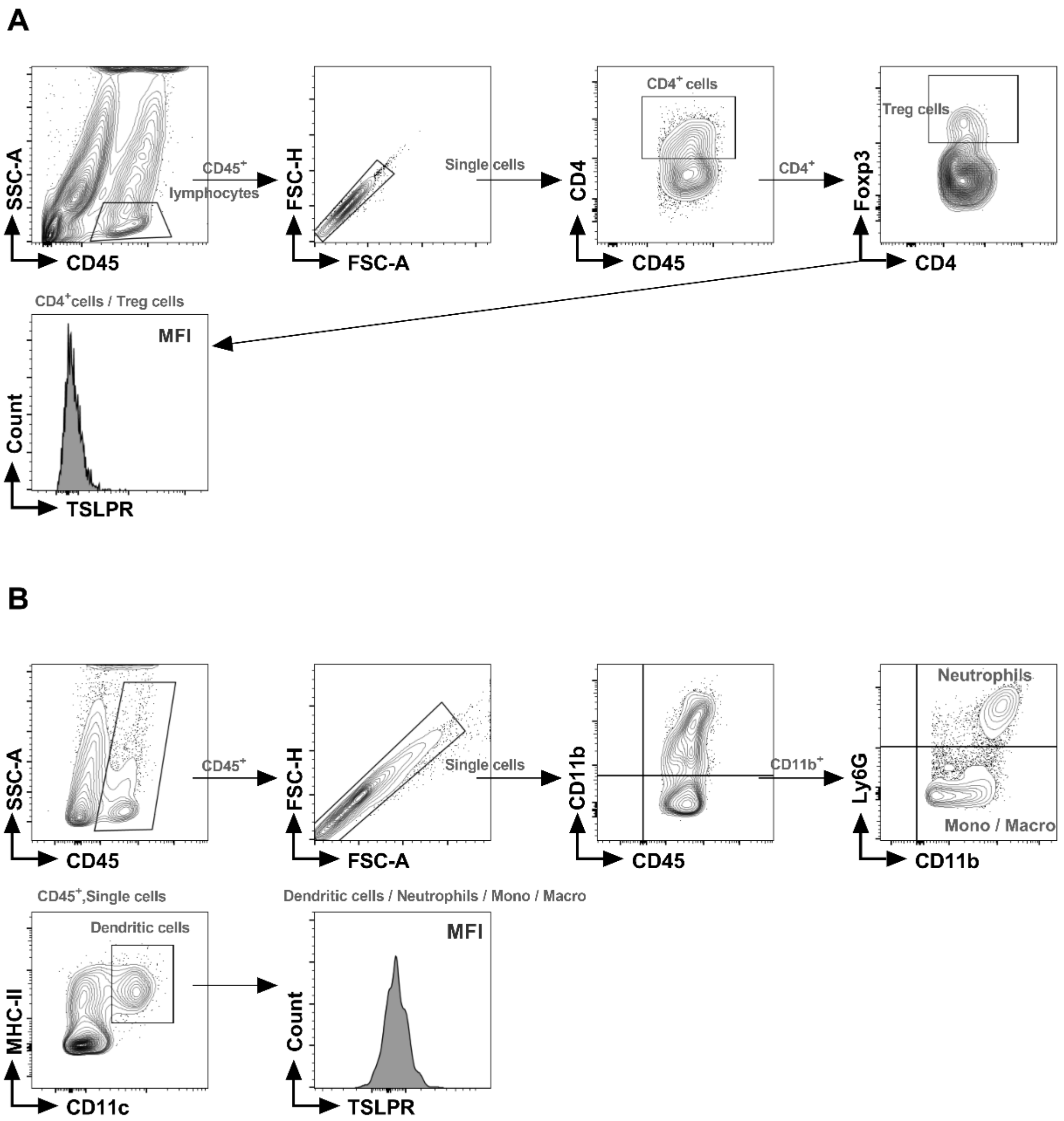

Supplementary Figure 2. Thymic stromal lymphopoietin receptor (TSLPR) distribution in immune cells of the mouse heart and spleen on day 7 post-MI. (A) Representative flow cytometric images of the gating strategies for $CD45^+CD4^+$ T cells and $CD45^+CD4^+Foxp3^+$ regulator T (Treg) cells in the heart. **(B)** Representative flow cytometric images of the gating strategies for $CD45^+CD11b^+Ly6G^+$ neutrophils, $CD45^+CD11b^+Ly6G^-$ monocytes/macrophages (Mono/Macro) and $CD45^+CD11c^+MHC\ II^+$ dendritic cells in the heart.

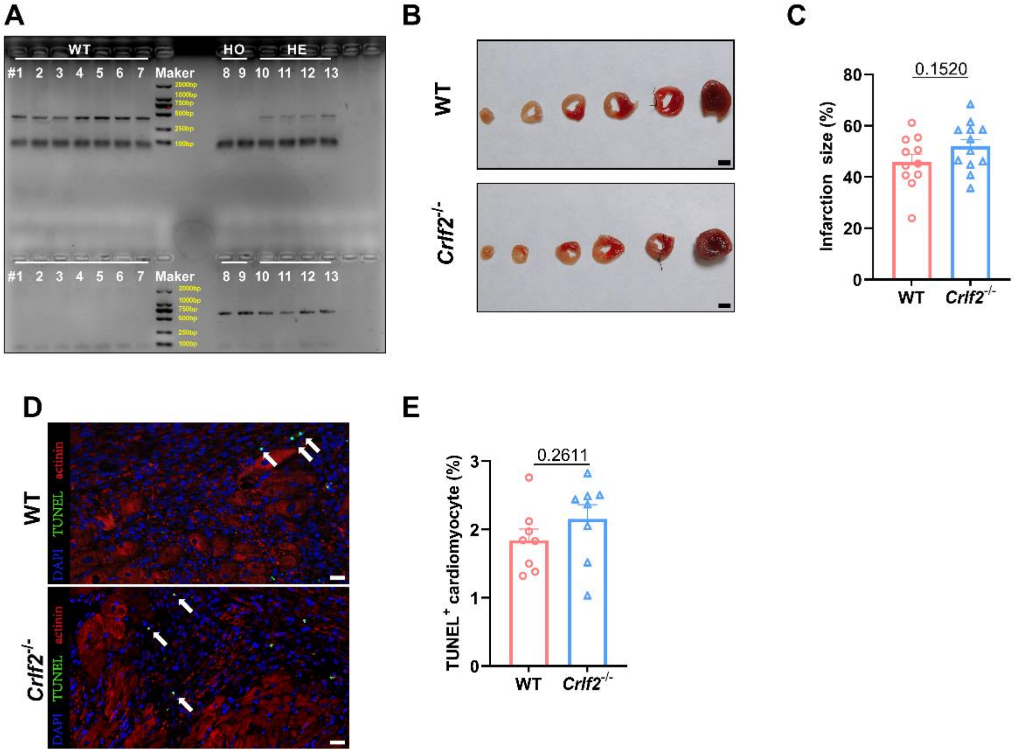

Supplementary Figure 3. Genotyping of TSLPR knockout (*Crlf2*$^{-/-}$) mice and histological staining of heart tissue. (A) Genotyping results; samples were taken from the mouse tail. The upper band in the figure represents the wild-type fragment (410 bp), and the lower band corresponds to the knockout fragment (640 bp). Using this genotyping system, mice 1–7 were determined to be wild type (WT), mouse 8–9 was homozygous *Crlf2*$^{-/-}$(HO), and mice 10–13 were heterozygous *Crlf2*$^{+/-}$ (HE). **(B and C)** Representative 2,3,5-triphenyl tetrazolium chloride staining (TTC) images (scale bar: 2 mm) and quantitative analysis in the mouse heart on day 1 post-MI (n = 11–12 per group). **(D and E)** Representative images showing TUNEL$^+$ staining in the border zone on day 7 post-MI (scale bar: 20 μm) and quantification of the TUNEL$^+$ cardiomyocyte (n = 8 per group). Data are presented as the mean ± SEM. Data in C and E were analyzed using unpaired t-test.

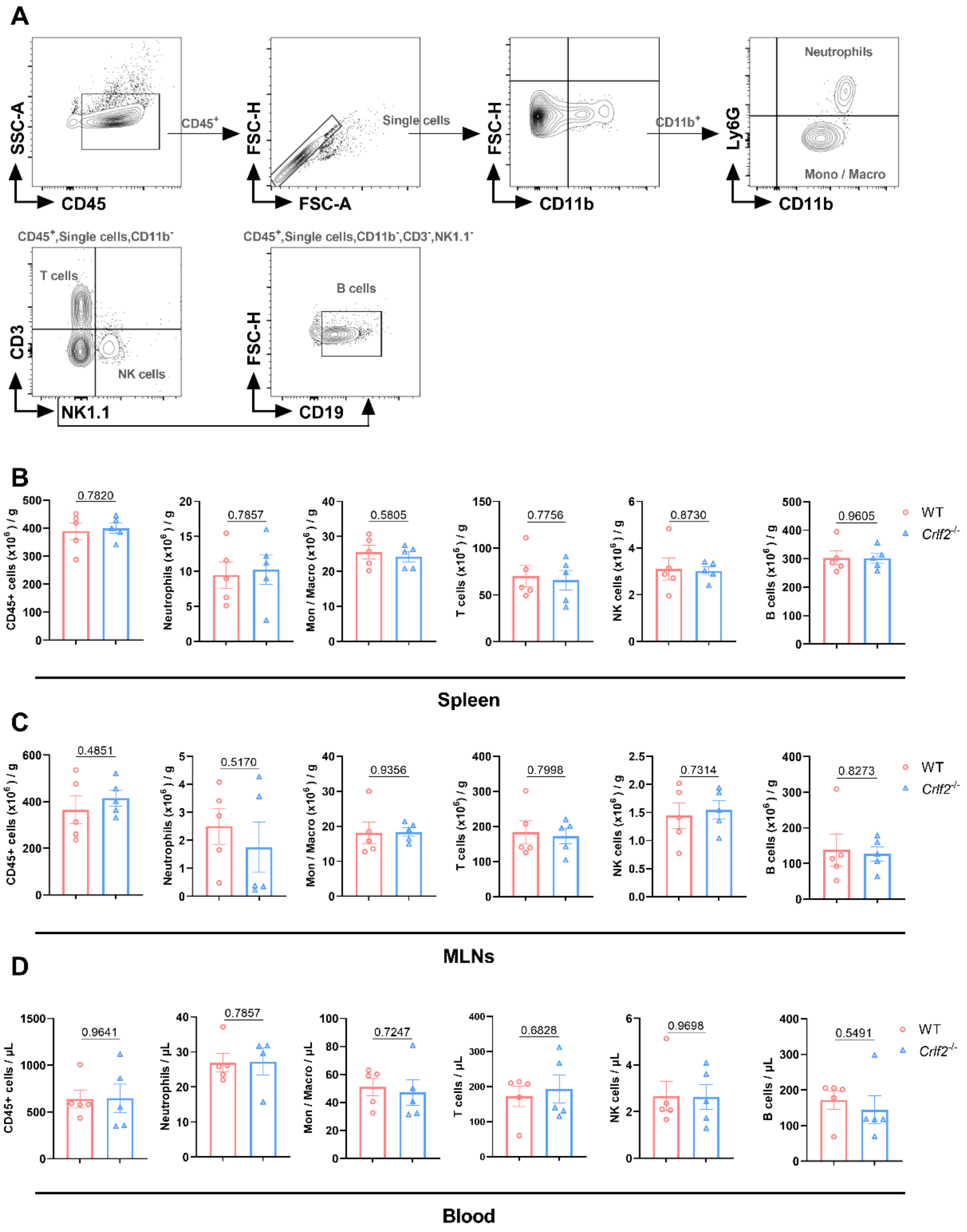

Supplementary Figure 4. Baseline levels of immune cells in the spleen, mediastinal lymph nodes (MLNs), and blood compared between wild-type (WT) and TSLPR knockout (*Crlf2*$^{-/-}$) mice. (A) The gating strategies for total CD45^{+} cells, CD45^{+}CD11b^{+}Ly6G^{+} neutrophils, CD45^{+}CD11b^{+}Ly6G^{-} monocytes/macrophages (Mono/Macro), CD45^{+}CD11b^{-}CD3^{+} T cells, CD45^{+} CD11b^{-}NK1.1^{+} natural killer (NK) cells, and CD45^{+}CD11b^{-}CD19^{+} B cells. **(B–D)** The absolute numbers of above cells were quantitatively determined (n = 5 per group). Data are presented as the mean ± SEM. For data in B–D, unpaired t-test was performed.

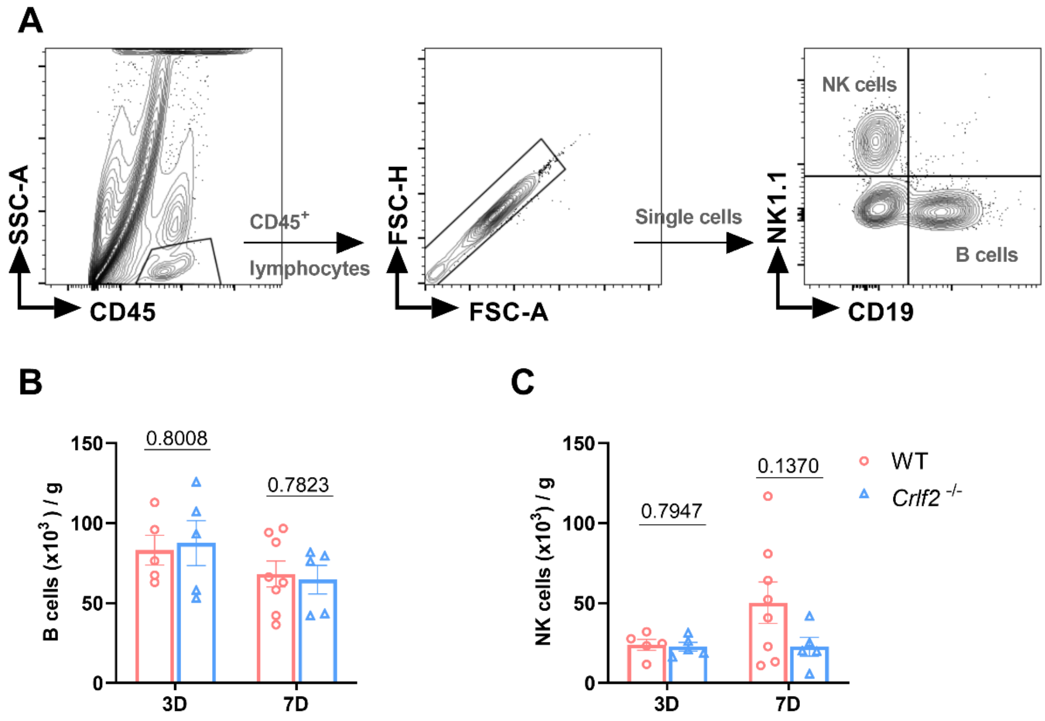

Supplementary Figure 5. The absence of the thymic stromal lymphopoietin (TSLP)/TSLP receptor (TSLPR) signaling pathway does not affect the infiltration of B cells and NK cells in cardiac tissue after MI. (A) The gating strategies employed in identifying infiltrated $CD45^+CD19^+$ B cells and $CD45^+NK1.1^+$ natural killer (NK) cells in the heart on day 3 and 7 post-MI. **(B and C)** The absolute numbers of the above cells were determined (n = 5–8 per group). Data are presented as the mean ± SEM. For data in B–C, unpaired t-test was performed.

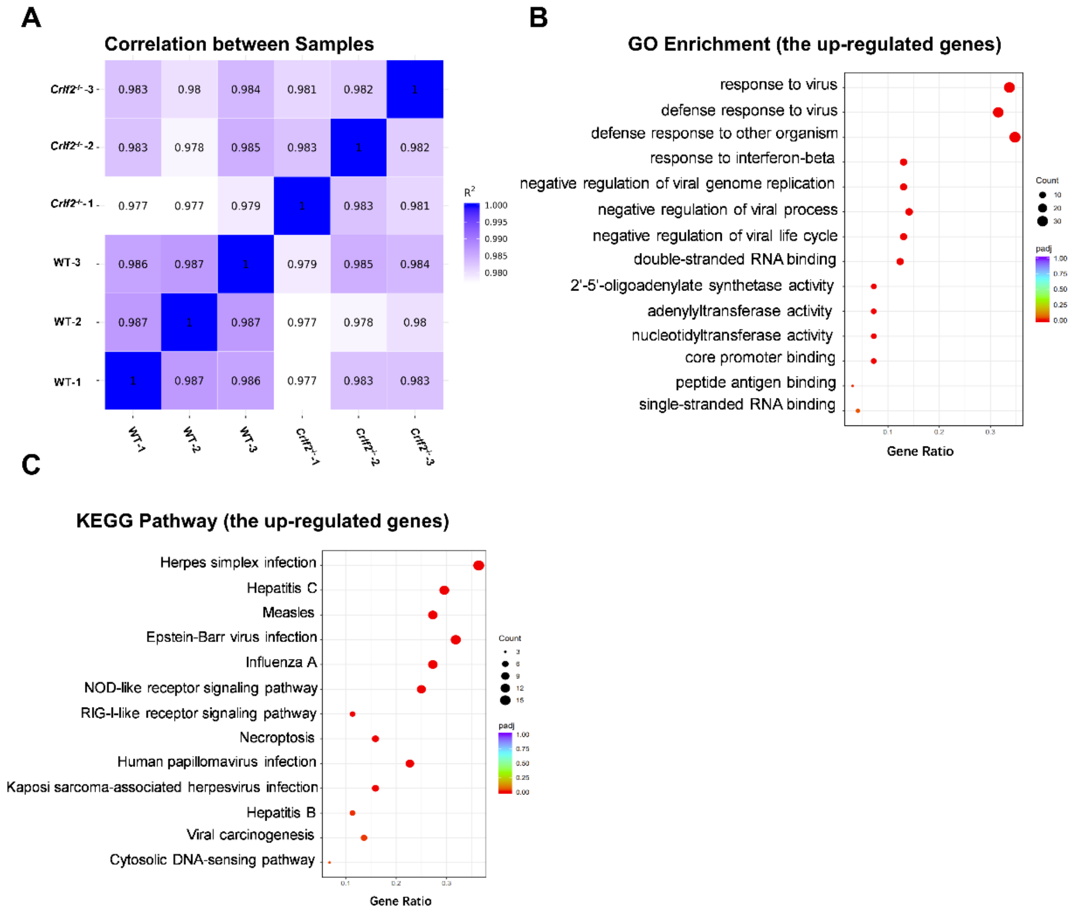

Supplementary Figure 6. Impact of the thymic stromal lymphopoietin (TSLP)/TSLP receptor (TSLPR) signaling pathway on the transcriptomic profile of the infarcted heart. (A) Correlation analysis between wild-type (WT) and TSLPR knockout (*Crlf2*$^{-/-}$) mice (n = 3 per group). **(B and C)** Gene Ontology (GO) enrichment and Kyoto Encyclopedia of Genes and Genomes (KEGG) pathway analyses of the upregulated genes in *Crlf2*$^{-/-}$ mice.
